# Modulating electrolyte structure for ultralow temperature aqueous zinc batteries

**DOI:** 10.1038/s41467-020-18284-0

**Published:** 2020-09-08

**Authors:** Qiu Zhang, Yilin Ma, Yong Lu, Lin Li, Fang Wan, Kai Zhang, Jun Chen

**Affiliations:** Key Laboratory of Advanced Energy Materials Chemistry (Ministry of Education), Renewable Energy Conversion and Storage Center (RECAST), College of Chemistry, Nankai University, Tianjin, 300071 China

**Keywords:** Batteries, Batteries

## Abstract

Rechargeable aqueous batteries are an up-and-coming system for potential large-scale energy storage due to their high safety and low cost. However, the freeze of aqueous electrolyte limits the low-temperature operation of such batteries. Here, we report the breakage of original hydrogen-bond network in ZnCl_2_ solution by modulating electrolyte structure, and thus suppressing the freeze of water and depressing the solid-liquid transition temperature of the aqueous electrolyte from 0 to –114 °C. This ZnCl_2_-based low-temperature electrolyte renders polyaniline||Zn batteries available to operate in an ultra-wide temperature range from –90 to +60 °C, which covers the earth surface temperature in record. Such polyaniline||Zn batteries are robust at –70 °C (84.9 mA h g^−1^) and stable during over 2000 cycles with ~100% capacity retention. This work significantly provides an effective strategy to propel low-temperature aqueous batteries via tuning the electrolyte structure and widens the application range of temperature adaptation of aqueous batteries.

## Abstract (web summary)

Rechargeable aqueous batteries are promising for potential large-scale energy storage due to their high safety and low cost. Here the authors analyse a zinc chloride based low-temperature electrolyte for improving practicability of the aqueous batteries.

## Introduction

Electrochemical energy storage technologies are of significance for reserve and conversion of renewable natural resources^1–4^. Rechargeable aqueous Zn batteries have been considered as a promising candidate for large-scale energy storage due to the high safety, materials abundance, and the intrinsic merits of Zn in low redox potential (–0.76 V vs. standard hydrogen electrode) and high theoretical capacity (820 mA h g^−1^)^5–12^. However, the aqueous electrolyte owns narrow liquid-state temperature range. When temperature substantially decreases, common aqueous electrolyte easily gets frozen due to the high freezing point of water, which limits the mobility of ions and the wettability of electrolyte toward electrode^13^, resulting in deteriorative electrode-electrolyte interphase. However, there are abundant renewable natural resources in severely cold regions, and most of energy storage devices cannot endure low temperature^14,15^. Therefore, it is a priority for preventing the electrolyte from solidifying at subzero conditions and constructing low-temperature aqueous Zn batteries (LTZBs).

The freezing process is an intricate rearrangement from orderless water to ordered ice, which is driven by forming extra hydrogen bonds (H-bonds)^16–20^. Up to now, a few strategies have been proposed to suppress the freeze of water, such as forming eutectic with organics^21,22^ and anti-freezing hydrogel^23,24^. Though the feasibility of LTZBs has been demonstrated at –20 °C^23,24^, the ultimate low temperature for aqueous Zn batteries is still unknown and unreached. The batteries based on the organic electrolyte with low-freezing-point solvent, such as liquefied CO_2_/fluoromethane gas^25^, ethyl acetate^26^, and perfluorinated ether^27^, can easily reach the ultralow operation temperature of –60, –70 and –85 °C, respectively. However, the solvent of aqueous Zn electrolyte is fixed as water, which has a high freezing point of 0 °C at standard atmospheric pressure^28^. Despite the introduction of organics can suppress the freeze of water, it reduces the ionic conductivity of electrolyte (0.11 mS cm^−1^ at –50 °C) and thus restricts the lowest operation temperature of batteries up to –50 °C^21^. In addition, the organic electrolyte is generally evaporable and inflammable. Therefore, developing organics-free ultralow-temperature aqueous electrolyte with superior electrochemical performance is still needed. Metal ions possess strong electrostatic interaction with a dipolar water molecule and can significantly break the original H-bond network^29^, which renders the feasibility of regulating the freezing point of water by modulating H-bonds. ZnCl_2_ is one of the most soluble inorganic salts and can reduce H-bonds in water sharply with high concentration. While ‘water in salt’ (WIS) strategies widen the electrochemical window of electrolyte^30–36^, the further expanding WIS on the broad operating temperature range especially at ultralow temperature is still deficient. Thus, it is considered that how to modulate electrolyte structure to achieve ultralow solid-liquid transition temperature (T_t_) and obtain the relationship between electrolyte concentration and T_t_. In addition, the evolution of the electrolyte structure, as well as the thermodynamic transition properties with concentration, should be studied deeply by multi-perspective approach.

In this work, we aim at ZnCl_2_-based aqueous electrolyte and explore the relationship among ZnCl_2_ concentration (${\mathrm{C}}_{{\mathrm{ZnCl}}_2}$), electrolyte structure (including H-bonds and ion interactions) and T_t_. The main discovery is that the T_t_ gradually reduces as the ${\mathrm{C}}_{{\mathrm{ZnCl}}_2}$ increases dominated by the breakage of H-bonds from 1 to 7.5 m (mol kg^−1^) electrolyte, while the T_t_ increases at higher ${\mathrm{C}}_{{\mathrm{ZnCl}}_2}$ (>7.5 m) because of the enhanced ion interactions. Thus, the T_t_ and ${\mathrm{C}}_{{\mathrm{ZnCl}}_2}$ present a V-shape relationship. The lowest T_t_ of –114 °C is achieved at 7.5 m ZnCl_2_ electrolyte, and this electrolyte exhibits a high ionic conductivity (1.79 mS cm^−1^ at –60 °C) and good compatibility with Zn in an ultra-wide temperature range of −100~+60 °C. Furthermore, polyaniline (PANI)||Zn batteries with this low-temperature electrolyte (LTE) shows high tolerance at an extremely low temperature of −90 °C and stable cycling performance (84.9 mA h g^−1^, ~2000 cycles) at −70 °C. This work will broaden the design philosophy of antifreezing electrolyte and promote the wide-temperature large-scale energy storage adopting aqueous batteries.

## Results

### Low-T_t_ solution design

**Fig. 1:**
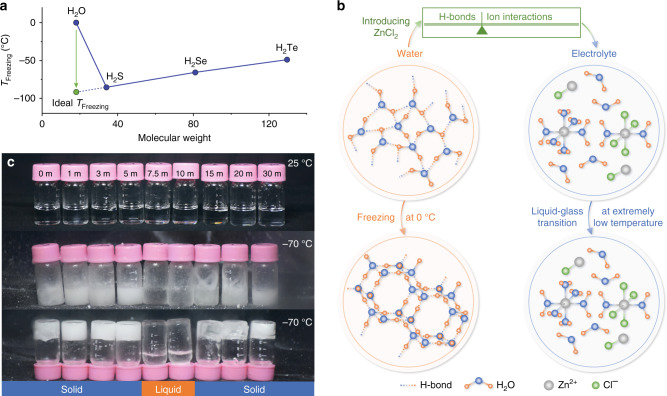
The schematic of the low-T_t_ solution design. **a** The freezing point of chalcogen hydrides. H_2_O visibly deviates from the law between the freezing point and molecular weight. The ideal freezing point is marked in the extended line. **b** The schematic of the structure evolutions of water and electrolyte, and the design of low-T_t_ solution. Original water network linked by H-bonds can easily transform to ice network at 0 °C. After adding ZnCl_2_, the H-bond network is broken by the strong interaction between ions and water, while the ion interactions are enhanced. By balancing the H-bonds and ion interactions for modulating T_t_, the electrolyte at critical ${\mathrm{C}}_{{\mathrm{ZnCl}}_2}$ can be operated at extremely low temperature. **c** The optical photographs of different ${\mathrm{C}}_{{\mathrm{ZnCl}}_2}$ electrolyte at 25 and −70 °C. At −70 °C, the electrolyte owning low/high ${\mathrm{C}}_{{\mathrm{ZnCl}}_2}$ is solid, while the moderate ${\mathrm{C}}_{{\mathrm{ZnCl}}_2}$ (7.5–10 m) electrolyte remains liquid.

H_2_O contains partially positively charged H atoms and partially negatively charged O atom. The H-bond (O–H···O) is formed between the O atom and the H atom of neighboring H_2_O via electrostatic interaction mainly^37^. Deviating significantly from the law of chalcogen hydrides (Fig. 1a), water owns the illogically high freezing point owing to its abundant H-bonds. Below 0 °C, water can easily transform into ice, accompanying with the formation of extra 0.52 H-bonds per H_2_O (Supplementary Note 1). Thus, destructing the H-bonds in water would enlarge the transformation energy gap between water and ice in thermodynamics. In addition, ice nucleation, as the initial stage of freeze, relies on the water molecules with tetrahedrally coordinated structure growing into stacked hexagonal sequences^17^ (Supplementary Fig. 1). Thus, regulating the H-bond quantity and reducing the highly H-bound water molecules can validly suppress the freeze of water in kinetic pathway.

With multiple-valence charge and small radius, Zn^2+^ owns a large electric field, and generates strong electrostatic interactions with the dipolar water molecule, and thus rearranges the coordination structures of water molecules around metal ions. The O atoms are confined by metal ions via hydration and hardly involved in the formation of H-bonds, resulting in significantly reduced H-bond amount. In principle, as the metal-salt concentration ascends, more H-bonds in water are destroyed. Consequently, the electrolyte with reduced H-bonds is expected to remain in liquid state at subzero temperature. However, introducing a mass of metal salts is a double-edged sword. The physical characteristics of the solution are also plagued by such concentrated content. First, the strengthened interactions between cations and anions bring about high viscosity, leading to a detrimental process of mass transfer and high T_t_^36,38,39^. Meanwhile, the solubility of metal-salts decreases greatly with temperature reducing, and the tendency of expelling salt severely limits the liquid temperature range. Therefore, to achieve low-T_t_ solution, we introduced the highly soluble salt−ZnCl_2_ and gave consideration to both modulations of the H-bonds and cation-anion interactions, implemented the maximum inhibition of the liquid-solid transition by regulating ${\mathrm{C}}_{{\mathrm{ZnCl}}_2}$. As illustrated in Fig. 1b, the original H-bond structure of water is broken because of the strong dipole-dipole force between ionic species and water molecules, and Zn^2+^ solvation configurations emerge. This electrolyte is mainly composed of the water molecule with weak H-bond interaction, Zn(H_2_O)_2_Cl_4_^2–^, ZnCl^+^, and Zn(H_2_O)_6_^2+^. The weak H-bond reduces the freezing point of water, while the enhanced ion interactions raise the T_t_ of electrolyte. Thus, balancing the above two aspects can modulate T_t_, and the critical ${\mathrm{C}}_{{\mathrm{ZnCl}}_2}$ renders that this electrolyte can be operated at extremely low temperature, which is revealed by the liquid-state electrolyte with moderate ${\mathrm{C}}_{{\mathrm{ZnCl}}_2}$ at –70 °C (Fig. 1c).

### Electrolyte structures and solid-liquid transitions

**Fig. 2:**
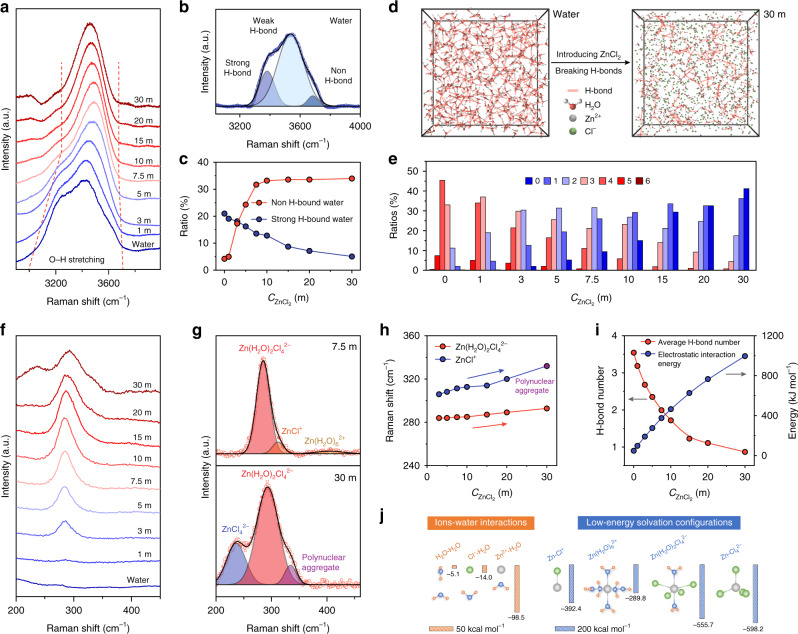
The electrolyte structures including H-bonds and ion interactions. **a** O–H stretching vibration of different ${\mathrm{C}}_{{\mathrm{ZnCl}}_2}$ electrolyte. **b** The fitted O–H stretching vibration representing water molecules with strong, weak and non H-bonds. **c** The proportion of strong H-bound water and non H-bound water. **d** The snapshot of the MD simulation of water and 30 m ZnCl_2_ electrolyte. The red line represents the H-bonds. **e** The proportion of the different H-bound water molecules. **f** The Raman peaks representing Zn^2+^ solvation configurations. **g** The fitted peaks of 7.5 and 30 m ZnCl_2_ electrolyte. **h** The peak value of Zn(H_2_O)_2_Cl_4_^2–^, ZnCl^+^ and polynuclear aggregate. **i** The H-bond number and the electrostatic interaction energies obtained by MD simulation. **j** The calculated interactions between ions and water, and the formation energies of Zn^2+^ solvation configurations.

Raman spectroscopy was performed to explore the evolutions of the H-bonds and the formations of the solvation configurations in the electrolyte with the increasing ${\mathrm{C}}_{{\mathrm{ZnCl}}_2}$. In Fig. 2a, b, O–H stretching vibration of water molecules (3000–3700 cm^−1^) shows an obvious wide peak, which is often convolved into three components. Based on previous reports^40,41^, the Raman peak gradually blueshifts as the H-bond interaction wanes, so the major peak wavenumbers located at ∼3230, ∼3450, and ∼3620 cm^−1^ correspond to the water molecules with strong, weak and non H-bonds, respectively. With the addition of ZnCl_2_, the peaks narrow to high frequency, indicating the decrease of strong H-bond proportion in the solution^42^. To further quantify the water in different H-bound states, the ratios are calculated based on the area of fitted peaks (Supplementary Fig. 2). In Fig. 2c, the strong H-bound water reduces while non H-bound water increases with the ${\mathrm{C}}_{{\mathrm{ZnCl}}_2}$, suggesting the progressive destruction of H-bond structure with salt concentration. The ^1^H nuclear magnetic resonance (NMR) was also utilized as a sensitive indicator to further study the H-bond network in the electrolyte^43,44^. As we can see, ^1^H chemical shifts to high field as ${\mathrm{C}}_{{\mathrm{ZnCl}}_2}$ owing to the breakage of the H-bonds among water molecules (Supplementary Fig. 3). Moreover, we simulated different ${\mathrm{C}}_{{\mathrm{ZnCl}}_2}$ electrolyte with molecular dynamics (MD). Intuitively, the snapshots of the simulated liquid phase show the massive reduction of H-bonds by inducing ZnCl_2_ (Fig. 2d). Specifically, to identify the local H-bond coordination of water molecules, the distribution of water molecules with different H-bond number was explored (Fig. 2e). In pure water, the water molecules with 4 H-bonds are dominant and constitute the water with tetrahedrally coordinated structure, which is decisive in the ice nucleation. When increasing the ${\mathrm{C}}_{{\mathrm{ZnCl}}_2}$, the H-bonds number of dominant water molecules reduces to 2 and even 0 at 7.5 and 30 m ${\mathrm{C}}_{{\mathrm{ZnCl}}_2}$ electrolyte, respectively. Thus, the decrease of 4 H-bound water molecules with ${\mathrm{C}}_{{\mathrm{ZnCl}}_2}$ increasing suppresses ice nucleation, which hinders the freeze of water from aspects of kinetics.

The solvation configurations of Zn^2+^ in the various ${\mathrm{C}}_{{\mathrm{ZnCl}}_2}$ electrolyte are also unveiled by Raman spectroscopy in the region of 200–450 cm^−1^ ^35,45^. As shown in Fig. 2f, the pure water shows no peaks around this range. In comparison, upon the introduction of ZnCl_2_, a new peak at 284.0 cm^−1^ appears and keeps increasing with the ${\mathrm{C}}_{{\mathrm{ZnCl}}_2}$, corresponding to the formation of Zn^2+^ solvation configurations. While the concentration increasing to the 30 m, the initial peak turns into two peaks, proving the new coordination way for Zn^2+^. In order to identify the species more precisely, we fitted the peaks and took 7.5 and 30 m ZnCl_2_ electrolyte as examples of moderate and high ${\mathrm{C}}_{{\mathrm{ZnCl}}_2}$ electrolyte (Fig. 2g and Supplementary Fig. 4). For 7.5 m ZnCl_2_ electrolyte, there are three peaks located at 284.9, 311.8 and 405.6 cm^−1^, representing Zn(H_2_O)_2_Cl_4_^2–^, ZnCl^+^ and Zn(H_2_O)_6_^2+^ respectively. For the almost saturated concentration of 30 m ${\mathrm{C}}_{{\mathrm{ZnCl}}_2}$, ZnCl_4_^2–^ and Zn–Cl polynuclear aggregate, of which peaks located at 236.4 and 334.3 cm^−1^, replaces Zn(H_2_O)_6_^2+^ and ZnCl^+^. The emerging Zn–Cl polynuclear aggregate is suggested like the melt/solid of ZnCl_2_^45^. Owing to the competition between Cl^–^ and water molecules for positions adjacent to Zn^2+^, the average number of coordinating water around Zn^2+^ is slashed and the water-free ZnCl_4_^2–^ and Zn–Cl polynuclear aggregate are formed with the ${\mathrm{C}}_{{\mathrm{ZnCl}}_2}$ increasing. The average coordinating numbers of Zn with O (H_2_O) and Cl are calculated based on MD simulation, demonstrating the dehydration of Zn^2+^ solvation configurations with ${\mathrm{C}}_{{\mathrm{ZnCl}}_2}$ increasing (Supplementary Fig. 5). More importantly, the Raman peak value of Zn(H_2_O)_2_Cl_4_^2–^ and ZnCl^+^ are exhibited in Fig. 2h and the blueshift of peaks unveil the enhanced ion interactions with increased ${\mathrm{C}}_{{\mathrm{ZnCl}}_2}$^38^, which is also demonstrated by the total electrostatic interaction potential energy among cations, anions and water molecules in MD simulation (Fig. 2i). As same as H-bond, the enhanced ion interactions will elevate the T_t_ of electrolyte. Inevitably, there is a critical ${\mathrm{C}}_{{\mathrm{ZnCl}}_2}$ contributing low H-bond and ion interactions, which may achieve the lowest T_t_ and apply to low-temperature electrolyte.

Toward revealing the mechanism of H-bond breakage and solvation configurations formation resulted from introducing ZnCl_2_, the interactions among ions and water were conducted by density functional theory (DFT) calculation. Dominated by electrostatic interaction, the H_2_O-H_2_O interaction (regarded as H-bond) exhibits binding energy of –5.1 kcal mol^−1^. Comparing to the water molecule with no charge, ions show stronger electrostatic and induction interaction, and thus higher binding energies^37^. The high binding energy of –98.5 kcal mol^−1^ between Zn^2+^ and H_2_O endows Zn^2+^ with the ability to break the H-bonds and reconstitutes the electrolyte structure with solvation configurations (Fig. 2j). The Zn^2+^ solvation configurations are also explored, including Zn(H_2_O)_2_Cl_4_^2–^, ZnCl^+^, Zn(H_2_O)_6_^2+^, and ZnCl_4_^2–^. The high formation energies imply that they are in a stable and low-energy state, which makes them more stable and difficult to dissociate into ions and water.

**Fig. 3:**
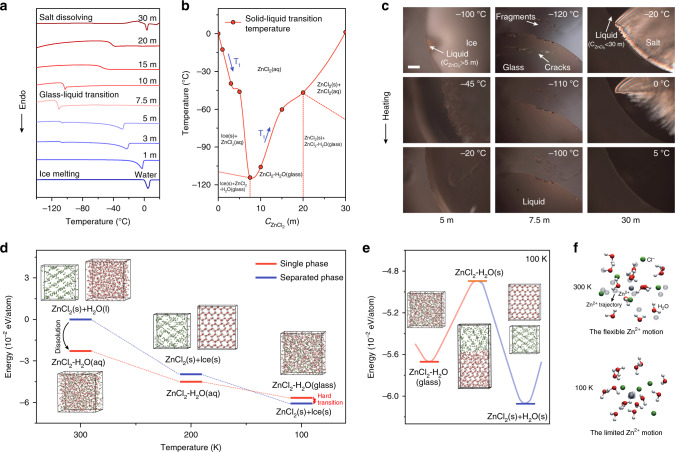
The properties of solid-liquid transitions and the mechanism of vitrification. Thermodynamic change and transition of different ${\mathrm{C}}_{{\mathrm{ZnCl}}_2}$ electrolyte at low temperature. **a** DSC test from −150 to 20 °C at heating rate of 5 °C min^–1^. **b** The major T_t_ verse ${\mathrm{C}}_{{\mathrm{ZnCl}}_2}$ and the phase composition of ZnCl_2_ solution at different temperature and concentration. **c** Polarizing microscope observation of 5, 7.5, and 30 m ZnCl_2_ electrolyte at around the respective T_t_. The scale bar is 200 μm. **d** The calculated energy profiles of the separated phase of ZnCl_2_ and H_2_O, and the single phase after ZnCl_2_ dissolved in H_2_O at 100~300 K. **e** The calculated transition state in the transformation from glass state to the separated crystalline state. **f** The Zn^2+^ motion trajectory in MD simulation at 100 and 300 K.

To demonstrate our design principle for the low-T_t_ solution, the characteristic and the temperature of the solid-liquid transitions of different ${\mathrm{C}}_{{\mathrm{ZnCl}}_2}$ electrolyte was explored by differential scanning calorimeter (DSC), which can specifically reveal the thermodynamic change accompanied by temperature^46,47^. Figure 3a shows the heat changes of different ${\mathrm{C}}_{{\mathrm{ZnCl}}_2}$ electrolyte by rising temperature from –150 to 20 °C. It is found that the electrolyte of different ${\mathrm{C}}_{{\mathrm{ZnCl}}_2}$ shows various solid-liquid transitions, including ice melting, glass–liquid transition, and salt dissolving. Generally, the ice melting and salt dissolving processes show sharp endothermic peaks and the glass–liquid transition shows up as an obvious step caused by the increased heat capacity^46^. The occurrence of vitrification results from the abundant existence of low-energy structure, which leads to the electrolyte trapped into the local minima energy^48,49^. Figure 3b clearly exhibits the V-shape relationship between the major T_t_ and ${\mathrm{C}}_{{\mathrm{ZnCl}}_2}$, and the lowest T_t_ of −114 °C is achieved at a critical concentration of 7.5 m. Below 7.5 m, the T_t_ is mainly dominated by reduced H-bonds. While above 7.5 m, the T_t_ increases with the ${\mathrm{C}}_{{\mathrm{ZnCl}}_2}$, because the T_t_ of this ${\mathrm{C}}_{{\mathrm{ZnCl}}_2}$ region is dominated by the enhanced ion interactions. At low ${\mathrm{C}}_{{\mathrm{ZnCl}}_2}$ of 1–5 m, the heating process contains two solid-liquid transitions—the minor process of glass–liquid transition below –100 °C and the major process of ice melting. In the ${\mathrm{C}}_{{\mathrm{ZnCl}}_2}$ range of 7.5–20 m, only glass–liquid transition occurs. When ${\mathrm{C}}_{{\mathrm{ZnCl}}_2}$ equals 30 m, the solid-liquid transitions include the minor process of glass–liquid transition below –60 °C and the major process of salt dissolving corresponding to the sharp endothermic peak at 1.2 °C. The specific T_t_ is summarized in Supplementary Table 1. To visualize those three transitions more intuitively, in situ polarizing microscope observation is applied in accordance with the isotropy and anisotropy of the electrolyte at different states^50^ (Fig. 3c). The boundary of the ice-liquid mixture is observed in the optical photograph of 5 m ZnCl_2_ electrolyte at –100 °C, demonstrating that the low ${\mathrm{C}}_{{\mathrm{ZnCl}}_2}$ electrolyte during cooling undergoes the freeze of water first and then the vitrification of remaining solution (whose ${\mathrm{C}}_{{\mathrm{ZnCl}}_2}$ is greater than that of the initial solution) at a lower temperature. In 7.5 m ZnCl_2_ electrolyte, there are fragments with sharp edges and cracks in the bulk glass at –120 °C, which caused by the liquid–glass transition and accompanying fracture of brittle glass. With the temperature increasing, the sharp edges of fragments become smooth and the cracks are restored, which indicates the electrolyte turns from brittle glass to mobile liquid. The absence of crystals at –120 °C proves the only formation of ZnCl_2_-H_2_O glass in 7.5 m ZnCl_2_ electrolyte. In high concentration of 30 m ZnCl_2_, the electrolyte shows the salt precipitation during cooling and turns into two phases of the solid ZnCl_2_ and the electrolyte of ${\mathrm{C}}_{{\mathrm{ZnCl}}_2}$<30 m, which undergoes the liquid–glass transition at a lower temperature. The phase composition of ZnCl_2_ solution at different temperature and concentration was drawn based on the DSC and polarizing microscope data.

To further unveil the unusual vitrification mechanism of ZnCl_2_-based electrolyte, we calculated the energy change of 7.5 m ZnCl_2_ electrolyte in the different states with the energy reference point of ZnCl_2_(s) and H_2_O(l) at 300 K. The high energy gap between ZnCl_2_(s)+H_2_O(l) separated phase and ZnCl_2_-H_2_O(aq) single-phase renders the easy dissolution of ZnCl_2_ at 300 K. When the temperature decreases to 200 K, the energy gap reduces dramatically owing to the freeze of H_2_O in the separated phase. With temperature further decreasing, the energy order is inverted, because the amorphous glass is metastable^51,52^ and owns higher thermodynamic energy than that in the form of crystallized ZnCl_2_ and ice. However, the transition from glass to crystal needs to overcome the energy barrier for the phase separation, which is interdicted owing to the limited thermal motion at the extremely low temperature (Fig. 3e, f). At 100 K, the selected Zn^2+^ was settled and can only move 0.5 Å at most around the origin, of which is 8.3 Å at 300 K (Supplementary Fig. 6). As a result, it is demonstrated by low-temperature Raman spectra that the water and ions are bound as similar as that in liquid state, and can hardly form crystallized ZnCl_2_ even at –150 °C (Supplementary Fig. 7), of which configuration reaches the minimum of system energy. The energy barrier and gap in the crystallization of glass imply that it occurs at relatively high temperature and is an exothermic process, which is proved by the cold crystallization of the ZnSO_4_-H_2_O glass at –52 °C (Supplementary Fig. 8).

### Low-temperature performance of electrolyte and Zn anode

**Fig. 4:**
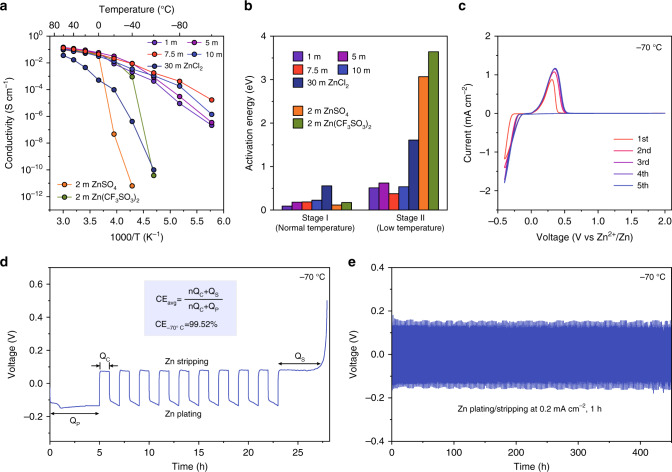
The electrochemcial performance of the LTE and Zn anode. **a** The ionic conductivities of the different electrolyte in the temperature range of –100~+60 °C. **b** The activation energies of ionic conduction in the different electrolyte at normal temperature and low-temperature stages. **c** CV curves of asymmetric Zn||Cu cell at –70 °C. **d** The voltage profiles of Zn plating/stripping in asymmetric Zn||Cu cell at –70 °C. **e** The cycling performance of symmetric Zn||Zn cell at –70 °C.

Apart from preventing electrolyte from freeze, the electrochemical performance of the LTE is also significant for operating LTZBs, especially with reference to the high ionic conduction and the good compatibility with Zn anode. To optimize and choose the appropriate LTE with superior ionic conduction at low temperature, the ionic conductivities of the different ${\mathrm{C}}_{{\mathrm{ZnCl}}_2}$ electrolyte (1, 5, 7.5, 10 and 30 m) and the common electrolyte (2 m ZnSO_4_ and 2 m Zn(CF_3_SO_3_)_2_ electrolyte) were tested in the temperature range of –100~+60 °C (Fig. 4a and Supplementary Table 2). Owing to the impeded ionic conduction of the fully frozen electrolyte at low temperature (Supplementary Fig. 9), 2 m ZnSO_4_ and 2 m Zn(CF_3_SO_3_)_2_ electrolyte exhibit the fast decay of ionic conductivities which are 6.41 × 10^–9^ and 3.79 × 10^–8^ mS cm^−1^ at –40 and –60 °C, respectively. As a comparison, the ZnCl_2_-based electrolyte shows relatively high ionic conductivity at –100~+60 °C, expect the WIS electrolyte of 30 m high concentration. Despite 1 and 5 m ZnCl_2_ electrolyte gets frozen at –12.6 and –46.0 °C respectively, they own high ionic conductivity at low temperature, resulting from the ionic conduction supported by the remaining concentrated electrolyte below their major T_t_. Meanwhile, the existence of solid ice impedes the ionic conduction and renders their ionic conductivities one-tenth less than the unfrozen 7.5 m ZnCl_2_ electrolyte at –80 °C. Though 10 m ZnCl_2_ electrolyte owns low T_t_ below –100 °C, it also shows faster reduction of ionic conductivity than 7.5 m ZnCl_2_ electrolyte. Based on the widened liquid-phase temperature range and relatively low concentration, the 7.5 m ZnCl_2_ electrolyte shows the high ionic conductivity of 1.79 mS cm^−1^ at –60 °C and 0.02 mS cm^−1^ even at –100 °C.

To precisely reveal the evolution of ionic conductivity with temperature, the activation energies of ionic conduction in electrolyte were calculated based on Arrhenius equation^3^. As shown in Fig. 4b, there are two stages including normal temperature stage and low-temperature stage, in which show different activation energies. The specific temperature range and activation energy for the different electrolyte are listed in Supplementary Table 3. Because of the exponential relationship between activation energy and ionic conductivity, the lower activation energy in ionic conduction implies the higher temperature independence of the electrolyte, which boosts its low-temperature performance. The activation energy in stage I clearly exhibits that the higher concentration of ZnCl_2_ electrolyte causes higher activation energy. At stage II, however, 7.5 m ZnCl_2_ electrolyte shows lowest activation energy of 0.374 eV caused by its unfrozen property above –100 °C, which leads to the highest low-temperature ionic conductivity among the above electrolyte. Despite 10 m ZnCl_2_ electrolyte is unfrozen at this temperature range, the higher concentration brings higher activation energy (0.536 eV), causing relatively low ionic conductivity. The partially frozen 1 and 5 m ZnCl_2_ electrolytes also show larger activation energy at stage II than the unfrozen 7.5 m ZnCl_2_ electrolyte. The 2 m ZnSO_4_ and Zn(CF_3_SO_3_)_2_ electrolyte of fully freeze show highest activation energies of 3.067 and 3.641 eV at stage II, respectively. Thus, the unfrozen property and relatively low concentration is required for LTE. The LTE design philosophy can be concluded as that utilizing lowest-concentration electrolyte with the premise of maintaining a liquid state at an ultralow temperature, which owns low activation energy and high ionic conductivity. As a result, we chose 7.5 m ZnCl_2_ electrolyte as LTE for further research on its electrochemical performance.

The asymmetric and symmetric Zn batteries were assembled and aimed to investigate the Zn compatibility of LTE. The cyclic voltammetry (CV) curve of asymmetric Zn||Cu batteries show the reversible redox reaction of Zn plating/stripping (Fig. 4c and Supplementary Fig. 10), and the high current density of 1.8 mA cm^−2^ can be achieved at –70 °C. For accurately quantifying the reversibility of Zn plating/stripping in LTE, coulombic efficiency (CE) of asymmetric Zn||Cu batteries were tested in common approaches^53,54^. The high average CE of 97.93 and 99.52% is achieved at 25 and –70 °C, respectively, and the long-term Zn plating/stripping are obtained (Fig. 4d and Supplementary Fig. 11). The high reversibility can be ascribed to the more Zn^2+^ solvation structure and less free solvent caused by the electrolyte of higher concentration^35,36^ (Supplementary Fig. 12). In addition, the symmetric Zn||Zn cells based on 2 m ZnSO_4_, Zn(CF_3_SO_3_)_2_ electrolyte and LTE were tested (Supplementary Fig. 13). The batteries with 2 m ZnSO_4_ and Zn(CF_3_SO_3_)_2_ electrolyte show sharply increased overpotential and battery failure due to the fully frozen electrolyte. Despite the symmetric battery using LTE shows enlarged overpotential owing to the reduced ionic conductivity and sluggish Zn plating/stripping kinetics at low temperature, it remains working at –90 °C, shows robust temperature stability and can withstand cycling at –70 °C for 450 h (Fig. 4e). The high ionic conductivity, good Zn compatibility and the wide electrochemical window (Supplementary Fig. 14) of LTE demonstrate its feasibility in the ultra-wide temperature range of –100~+60 °C.

### Batteries with LTE and PANI organic cathode

**Fig. 5:**
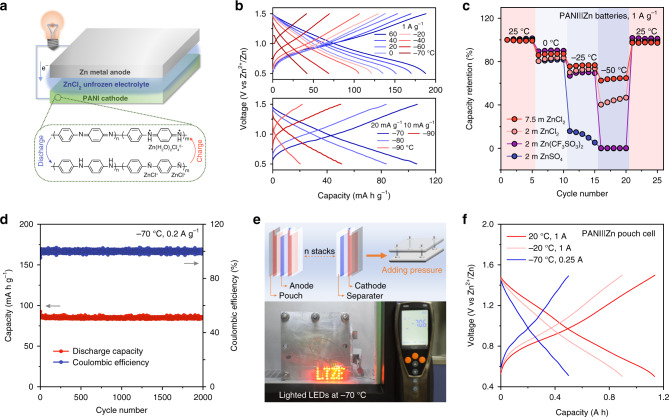
The energy storage mechanism and performance of PANI|LTE|Zn batteries. **a** The configurations and redox mechanism of PANI|LTE|Zn batteries. **b** Discharge–charge curves of PANI|LTE|Zn batteries at the temperature range of –90~+60 °C. **c** The comparison of batteries using common electrolyte and LTE in varying-temperature performance. **d** Cycling performance at –70 °C and 0.2 A g^–1^. **e** The schematic of assembled pouch cell and the lighted LEDs by two series-wound cells at –70 °C. **f** Discharge–charge curves of PANI|LTE|Zn pouch cell at 20, –20, and –70 °C.

The energy density of full batteries mostly depends on the electrochemical performance of cathode materials. However, Zn^2+^ with high charge exhibits the sluggish insertion kinetics for inorganic materials^55,56^, leading to the fast decay of capacity at low temperature. Recently, organic materials for low-temperature batteries have received attentions, owing to the charge storage mainly locating at surface groups and the high capacity independence of temperature^26,57,58^. Therefore, relying on the redox mechanism of the benzene/quinone structure transformation and the corresponding ions compensation, PANI was chosen for constructing LTZBs. The configurations and redox mechanism of PANI|LTE|Zn are shown in Fig. 5a.

The structure of obtained PANI was characterized by Raman spectroscopy^59–61^ (Supplementary Fig. 15). In order to demonstrate the benzene/quinone structure transformation mechanism and its consistency with temperature, in situ Raman spectroscopy is applied for PANI|LTE|Zn batteries at 20, –70, and –90 °C. The reversible quinone/benzene signal change of PANI cathode during the redox process at –70 °C, which is consistent in that at 20 °C and the extremely low temperature of –90 °C (Supplementary Note 2 and Fig. 16), demonstrates the high redox reversibility and cold tolerance of PANI cathode. With the internal structure transforming, cathode maintains charge balance by ions adsorption/desorption, which is proved by X-ray photoelectron spectroscopy and in situ electrochemical quartz crystal microbalance (Supplementary Figs. 17–18). Specifically, the ions compensation mechanism during discharging is expressed as Zn(H_2_O)_2_Cl_4_^2–^ desorption and H^+^/ZnCl^+^ hybrid adsorption (Supplementary Note 3), rendering the pseudo-capacitance energy storage in PANI|LTE|Zn batteries, which is verified by cyclic voltammetry (Supplementary Fig. 19). Despite the PANI cathode has the same redox mechanism at normal and low temperature, the slow mass transfer kinetics at low temperature renders the weakened redox peaks and enlarged electrochemical polarization.

Figure 5b shows the charge–discharge curves of PANI|LTE|Zn batteries ranged from 90 to +60 °C, which covers the recorded earth surface temperature from −89.2 °C (Death Valley, California, USA, 1913) to 56.7 °C (Vostok Station, Antarctica, 1983), and few batteries can operate in such severe temperature range (Supplementary Fig. 20). At the high current density of 1 A g^−1^, the capacities have a fast decay and the charge–discharge curves become near-triangular due to the reduced ionic transfer of the LTE and the enlarged electrochemical polarization. When the current reduces to 20 mA g^−1^, the capacity recovers to 106.2 mA h g^−1^ at –70 °C. The detailed rate performance at –70 °C is shown in Supplementary Fig. 21. Even if the temperature falls to –90 °C, the PANI|LTE|Zn batteries still remain working and exhibit capacity of 20.4 and 50.6 mA h g^−1^ at 20 and 10 mA g^−1^ respectively. As a comparison, the PANI||Zn batteries with 2 m ZnCl_2_, ZnSO_4_, and Zn(CF_3_SO_3_)_2_ electrolyte are hindered by the decreased operation temperature (Fig. 5c). The battery failure occurs successively at –25 °C for ZnSO_4_ electrolyte and –50 °C for Zn(CF_3_SO_3_)_2_ electrolyte, which is consistent with the symmetric Zn cell test. The battery based on 2 m ZnCl_2_ electrolyte shows relatively high capacity, because the electrolyte includes two phases at –50 °C: solid ice and ZnCl_2_ solution (${\mathrm{C}}_{{\mathrm{ZnCl}}_2}$ > 2 m, whose concentration maintains liquid at this temperature), and the liquid ZnCl_2_ solution sustains the battery operation. However, the ionic conduction impeded by solid ice results in the battery with low capacity retention (44.2%), demonstrating the necessity of the unfrozen electrolyte for LTZBs. The battery using unfrozen LTE shows superior low-temperature tolerance and high capacity retention of 64.7% at –50 °C. The cycling performance of the PANI|LTE|Zn batteries at –70 °C is shown in Fig. 5d, and long cycles (~2000) are achieved with the capacity retention of ~100% at 0.2 A g^−1^. Furthermore, the pouch cell of 1.15 A h is fabricated with stacked multilayer electrode. In Fig. 5e, the two pouch cells in series can power light-emitting diodes at –70 °C. The PANI|LTE|Zn pouch cell of 1.15 A h can retain the high capacity of 0.50 A h at –70 °C (43.4% capacity retention, Fig. 5f). This LTE-based pouch cell succeeds in the low-temperature tolerance and the energy densities of the pouch cell are estimated to be 97.9 and 42.6 Wh kg^−1^ at room temperature and –70 °C respectively based on the mass of active materials (The energy densities of 38.9 and 16.9 Wh kg^−1^ at room temperature and –70 °C are achieved based on the total mass of a cell, Supplementary Table 4). To broaden the application of LTE, in addition, the batteries coupled with the common inorganic cathode V_2_O_5_·1.6 H_2_O and LTE were also investigated. Though the low-temperature capacity retention of the V_2_O_5_·1.6 H_2_O|LTE|Zn batteries (45.9% at –50 °C) cannot come up to that of PANI|LTE|Zn batteries, it shows the higher capacity retention (68.6% at –25 °C) than that use 2 m ZnCl_2_, Zn(CF_3_SO_3_)_2_ and ZnSO_4_ electrolyte (62.8, 12.7 and 0.4% at –25 °C, respectively, Supplementary Fig. 22). As a result, the superior low-temperature electrochemical performance of PANI|LTE|Zn, V_2_O_5_·1.6 H_2_O|LTE|Zn batteries and the pouch cell demonstrate the high universality of LTE and the feasibility to meet the demand for large-scale application at the extremely cold conditions.

## Discussion

By balancing the strength of H-bonds and ion interactions in solution, we developed low-temperature aqueous electrolyte and presented the relationship among salt concentration, electrolyte structure, and thermodynamic transition property. The optimized ZnCl_2_-based electrolyte exhibits high ionic conductivity of 1.79 mS cm^−1^ at –60 °C, extremely low solid-liquid transition temperature (–114 °C), and good compatibility with Zn in an ultra-wide temperature range from –100 to +60 °C. Furthermore, PANI||Zn batteries using this aqueous electrolyte show an ultra-wide operation temperature range of –90~+60 °C (50.6, 105.6, 151.7, and 187.7 mA h g^−1^ at –90, –40, 20, and 60 °C respectively), and stable cycling performance (84.9 mA h g^−1^, ~2000 cycles) at –70 °C. This work not only demonstrates the universal application of ZnCl_2_-based low-temperature electrolyte, but also provides valuable insights and encouraging pathway towards propelling low-temperature aqueous electrolyte and batteries.

## Methods

### Materials

Zn foil (>99.99%) was purchased from Ailian of Tianjin. Zinc chloride (>99.99%) was purchased from Alfa Aesar. Other chemicals were purchased from Aladdin. The PANI was obtained by ammonium persulfate oxidizing aniline monomer in aqueous acid. In a typical synthesis^59^, 0.365 mL aniline was added into 15-mL HCl (1 M). Then 5-mL HCl (1 M) containing 0.228 g of ammonium persulfate was dropped into the above solution under an ice bath. The dark green sample was filtrated, washed with deionized water and ethanol and dried at 60 °C for 12 h successively. The V_2_O_5_·1.6 H_2_O was synthesized by a hydrothermal method. 0.91 g V_2_O_5_ powder, 12.5 mL H_2_O_2_ (30%) and 75-mL-deionized water were mixed and maintained at 190 °C for 10 h. After washed with deionized water, the product was obtained by freeze-drying.

### Characterizations

Raman spectroscopy for the electrolyte structure was conducted on Horiba LabRAM HR Evolution microscope. Raman spectroscopy for low temperature and in situ batteries was tested by confocal Thermo-Fisher Scientific DXR microscope. Both of them used a 532 nm excitation laser. DSC was carried out in METTLER TOLEDO DSC3 in the procedure of +25~−150 °C with a cooling rate of 10 K min^−1^, constant temperature for 2 mins and –150~+25 °C with a heating rate of 5 K min^−1^. The polarizing microscope was using Olympus BX51TRF. The refrigerating system for low-temperature characterizations is Linkam THMS600. NMR was characterized on Bruker ASCEND400. XPS was conducted on X-ray Photoelectron Spectrometer (Axis Ultra DLD) with an excitation source of Al K_α_ X-ray.

### Electrochemical measurements

The symmetric/asymmetric Zn battery using CR2032 coin cell was assembled with Zn metal electrode (Φ10 mm), 20-μL electrolyte, Celgard 3501 separator and Zn/Cu electrode. The PANI electrode was obtained by pressing the mixture of PANI, Ketjen black and polytetrafluoroethylene (PTFE) at a weight ratio of 6:3.5:0.5 on Ti mesh. The PANI||Zn and V_2_O_5_·1.6 H_2_O||Zn batteries based on the LTE and other Zn electrolyte were assembled in the same way. The discharge–charge profiles were conducted on LAND CT2000A. The CV and electrochemical window were tested by CHI660E. EQCM measurements were carried out on Princeton Applied Research QCM 922. An AT-cut Pt-coated quartz crystal with the resonance frequency of 9 MHz was coated with active material (PANI) and used as the working electrode. Zn foil was used as a counter and the reference electrode. The EQCM result was performed at room temperature in galvanostatic mode with low current of 10 μA. The Δmass of PANI electrode was calculated from the change in resonance frequency using the Sauerbrey equation. The low-temperature performance was tested in Meiling refrigerator DW-HW50 (–86~–10 °C) and the chamber of Suoyate WuXi (–70~60 °C). The extremely low temperature of –90 and –100 °C is achieved by melting solid n-Heptane and methanol (Supplementary Fig. 23).

### Computational methods

MD simulations for the H-bonds of different ${\mathrm{C}}_{{\mathrm{ZnCl}}_2}$ electrolyte was conducted on the GROMACS package^62^ with AMBER03 force field^63^. Water molecule was simulated with TIP4P model^64^. First, NVT run was performed at 298.15 K for 10 ns, and then NPT run was 70 ns long for ensuring the system equilibrium. The last 20 ns in NPT run was used for analysis. The calculation of H-bonds is based on the geometrical configuration that the distance of two O is <3.5 Å and the angle of O–H···O is <30°. Quantum chemistry calculations were performed using the Gaussian 16 software package. The B3LYP/6-311+G(d) for H, O, Cl and B3LYP/SDD for Zn were used for structure optimization as well as the energy calculation. The snapshot of MD simulation is produced by VMD software^65^.

## Supplementary information

Supplementary Information

## Data Availability

The data that support the findings of this study are available from the corresponding author upon reasonable request.
