# Supplementary material for: Modulating electrolyte structure for ultralow temperature aqueous zinc batteries
Source: Nat Commun. 2020 Sep 8;11:4463. doi: 10.1038/s41467-020-18284-0 (PMC7479594; doi:10.1038/s41467-020-18284-0)
Supplement: Supplementary file 1 — Supplementary Information [file 41467_2020_18284_MOESM1_ESM.pdf]

Supplementary Information

Modulating electrolyte structure for ultralow temperature aqueous zinc batteries

Zhang et al.

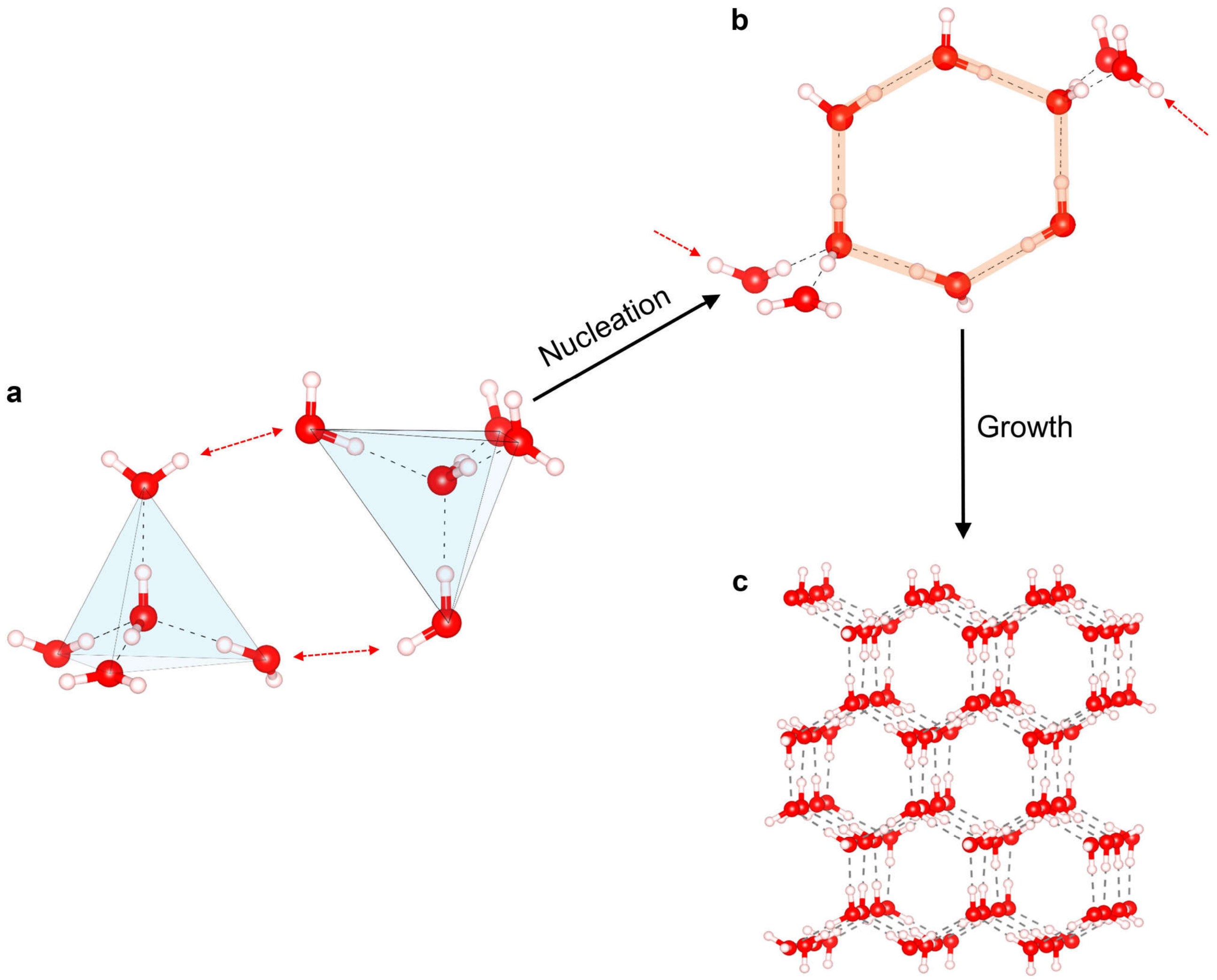

Supplementary Figure 1. The possible ice nucleation and growth mechanism. a, the adjacent two water molecules with tetrahedrally coordinated structure. b, the formed hexagonal sequences. c, the ice I_h phase by ice nuclei extending.

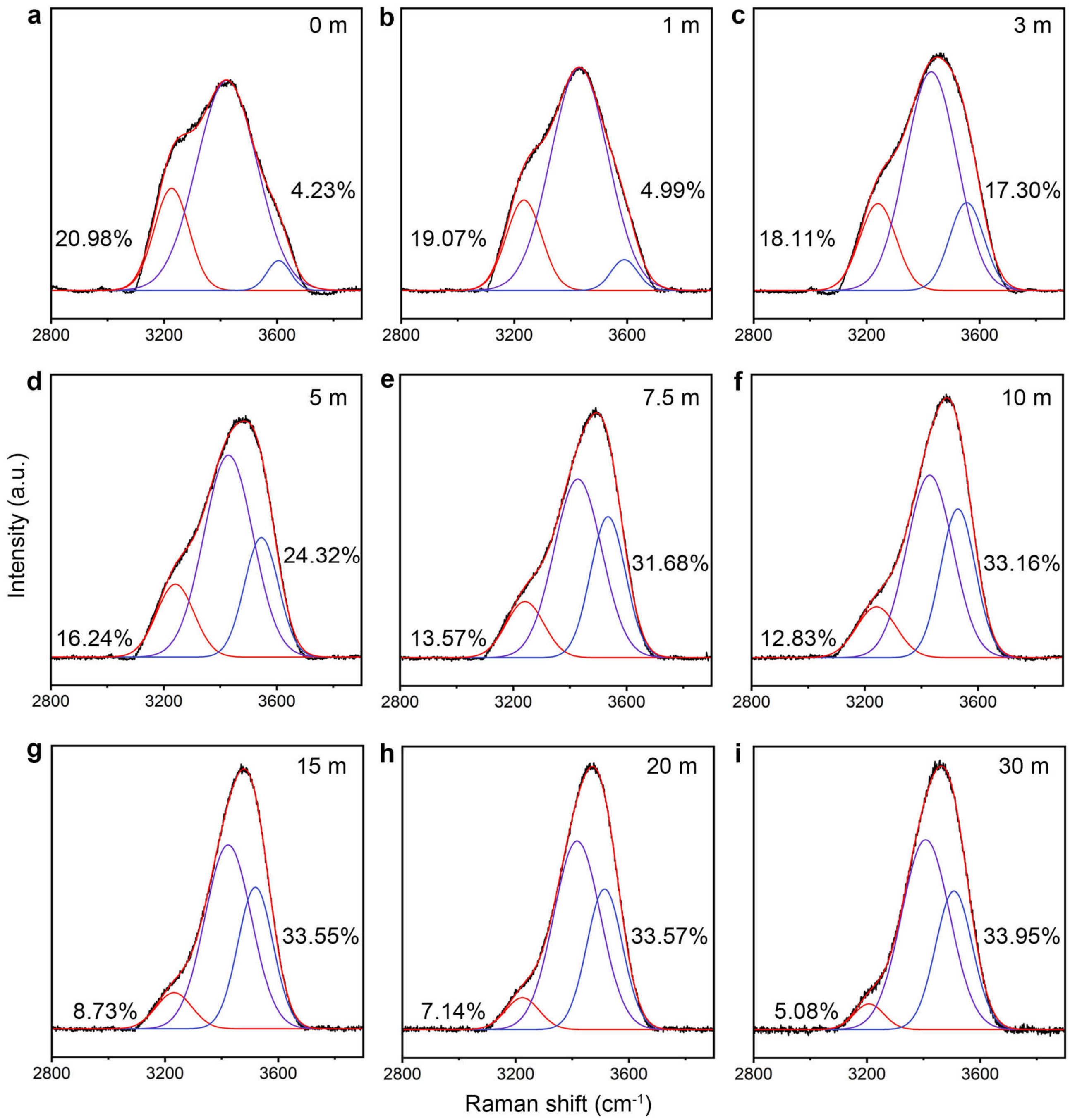

Supplementary Figure 2. a-i, The fitted peaks of Raman spectroscopy representing the O-H stretching vibration of water molecules in 0, 1, 3, 5, 7.5, 10, 15, 20 and 30 m C_{ZnCl_2} electrolyte, respectively.

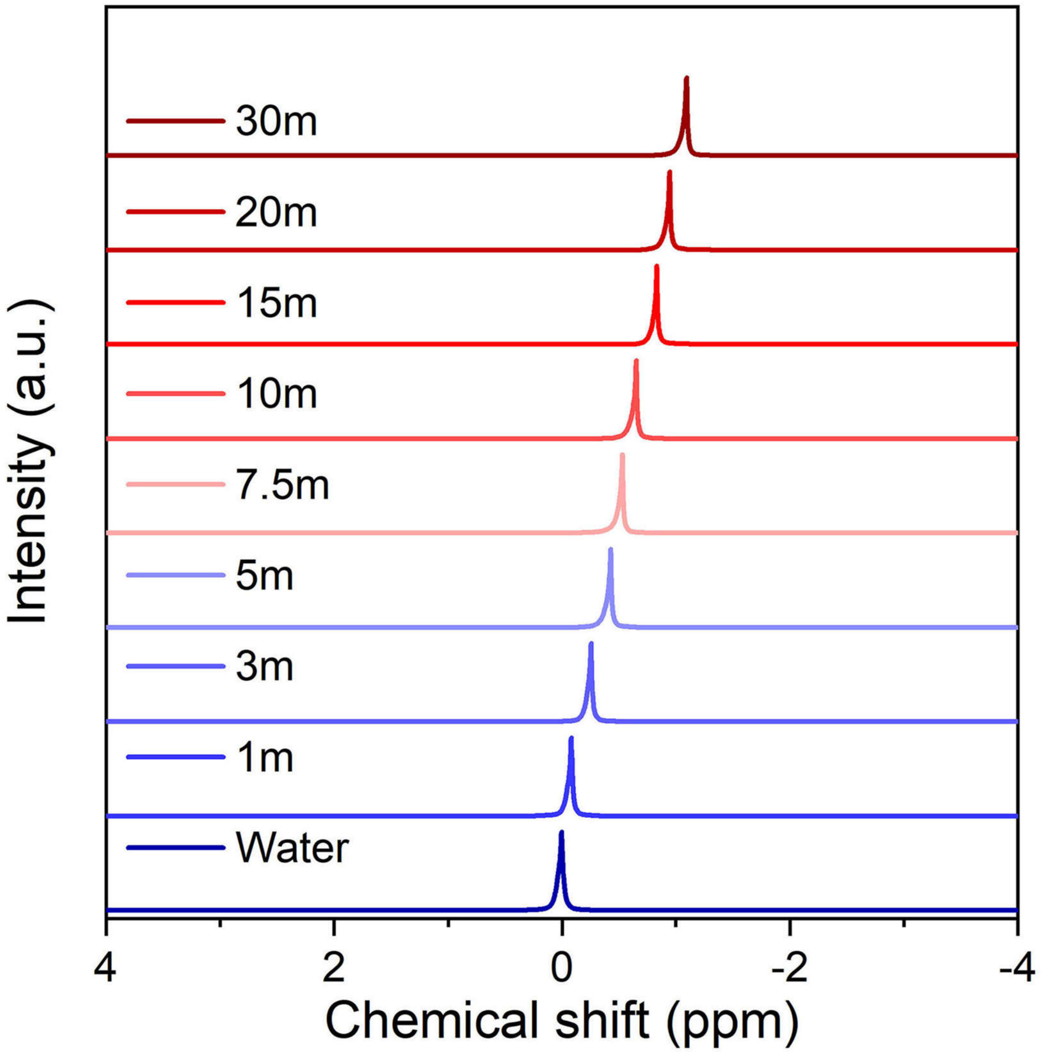

Supplementary Figure 3. ^{1}H chemical shift of water molecular in different C_{ZnCl_2} electrolyte. The NMR test for the electrolyte is on the base of the ^{1}H chemical shift of pure water. The ^{1}H shifts to high field with C_{ZnCl_2} increasing, indicating the enhancement of shielding effect which is caused by the reduction of H-bonds.

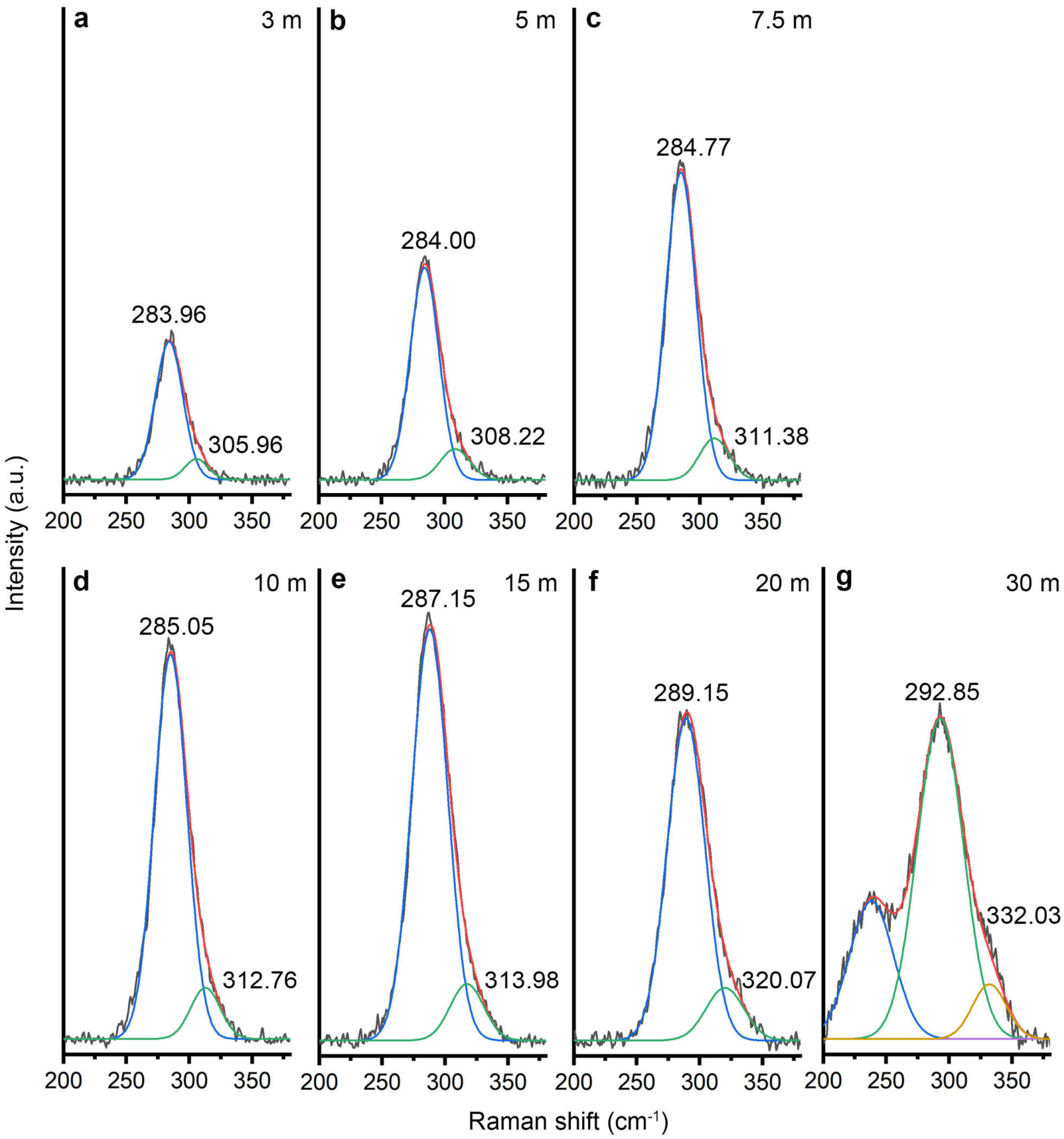

Supplementary Figure 4. a-g, The fitted peaks of Raman spectroscopy representing the solvation configurations of Zn^{2+} in 3, 5, 7.5, 10, 15, 20 and 30 m C_{ZnCl_2} electrolyte, respectively.

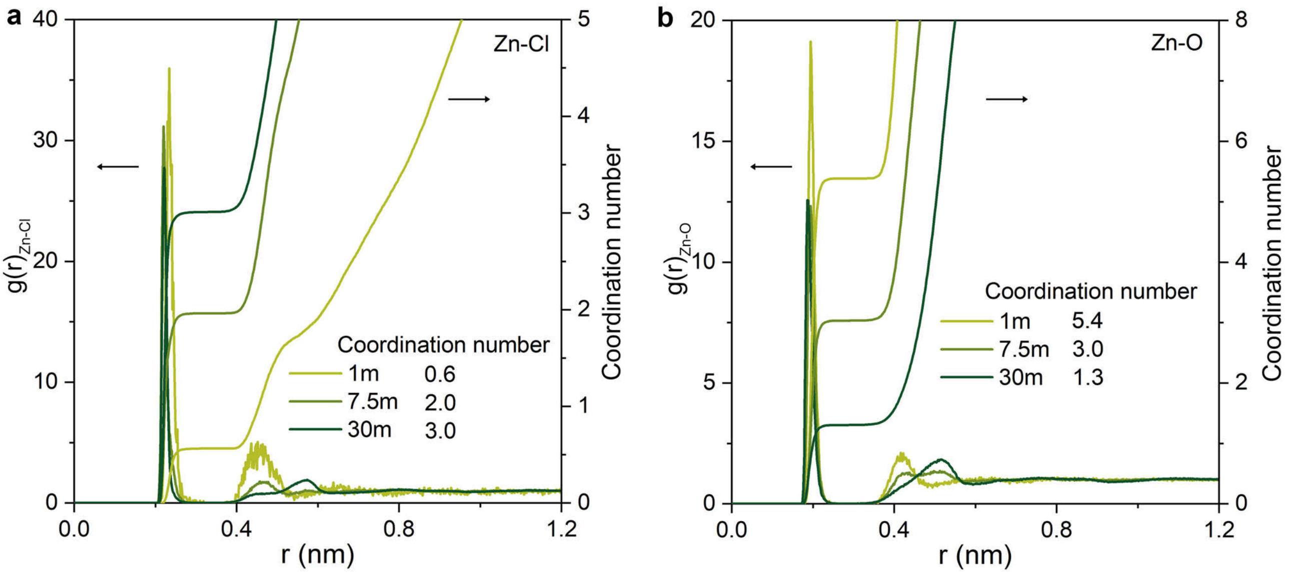

Supplementary Figure 5. The radial distribution function (RDF) and the corresponding average coordination number (N). a, The RDF and N between Zn^{2+} and Cl^{-}. b, The RDF and N between Zn^{2+} and O atom in H_2O. With the C_{ZnCl_2} increasing, the N of Zn-Cl increases, while the N of Zn-O decreases due to the reduced water molecules.

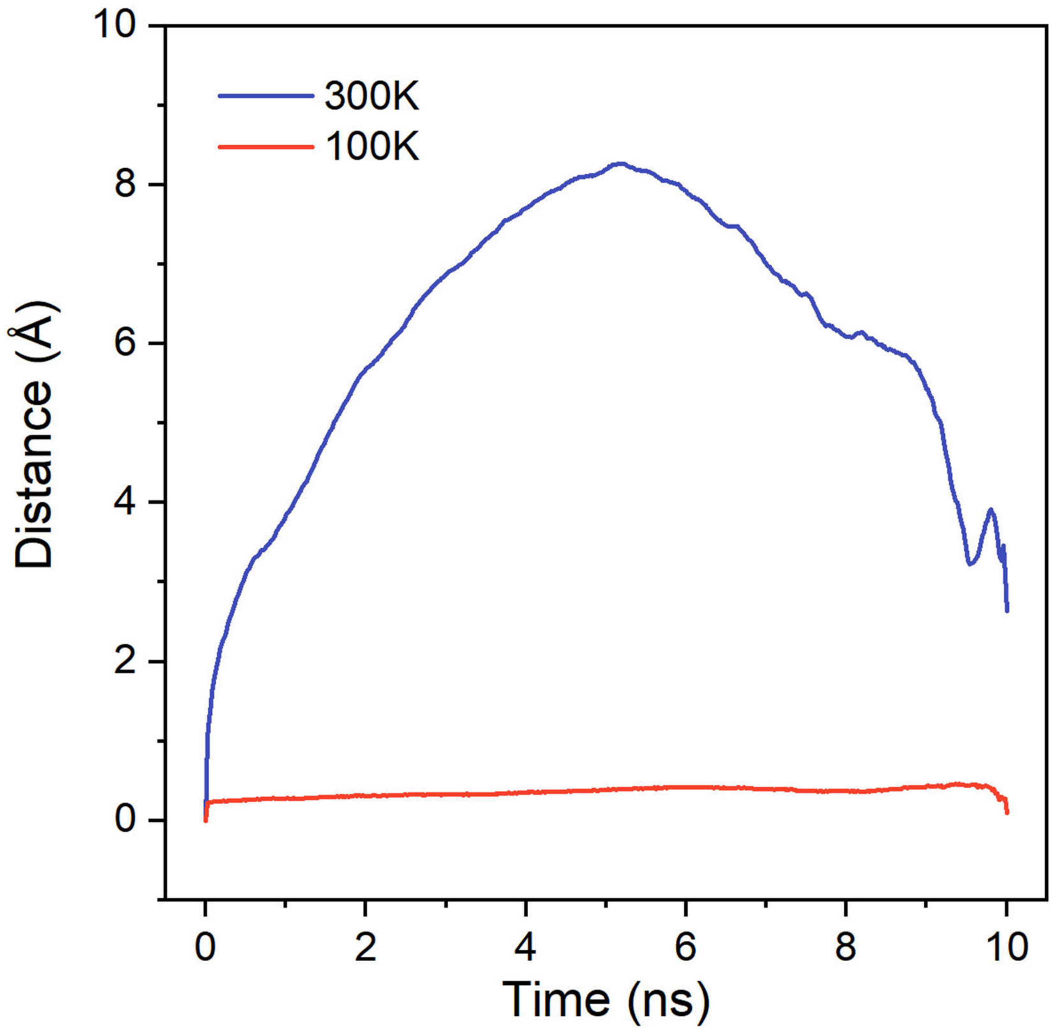

Supplementary Figure 6. Motion distance relative to the origin of selected Zn atom during the MD simulation. The longest motion distance at 100 and 300 K is 0.5 and 8.3 Å respectively.

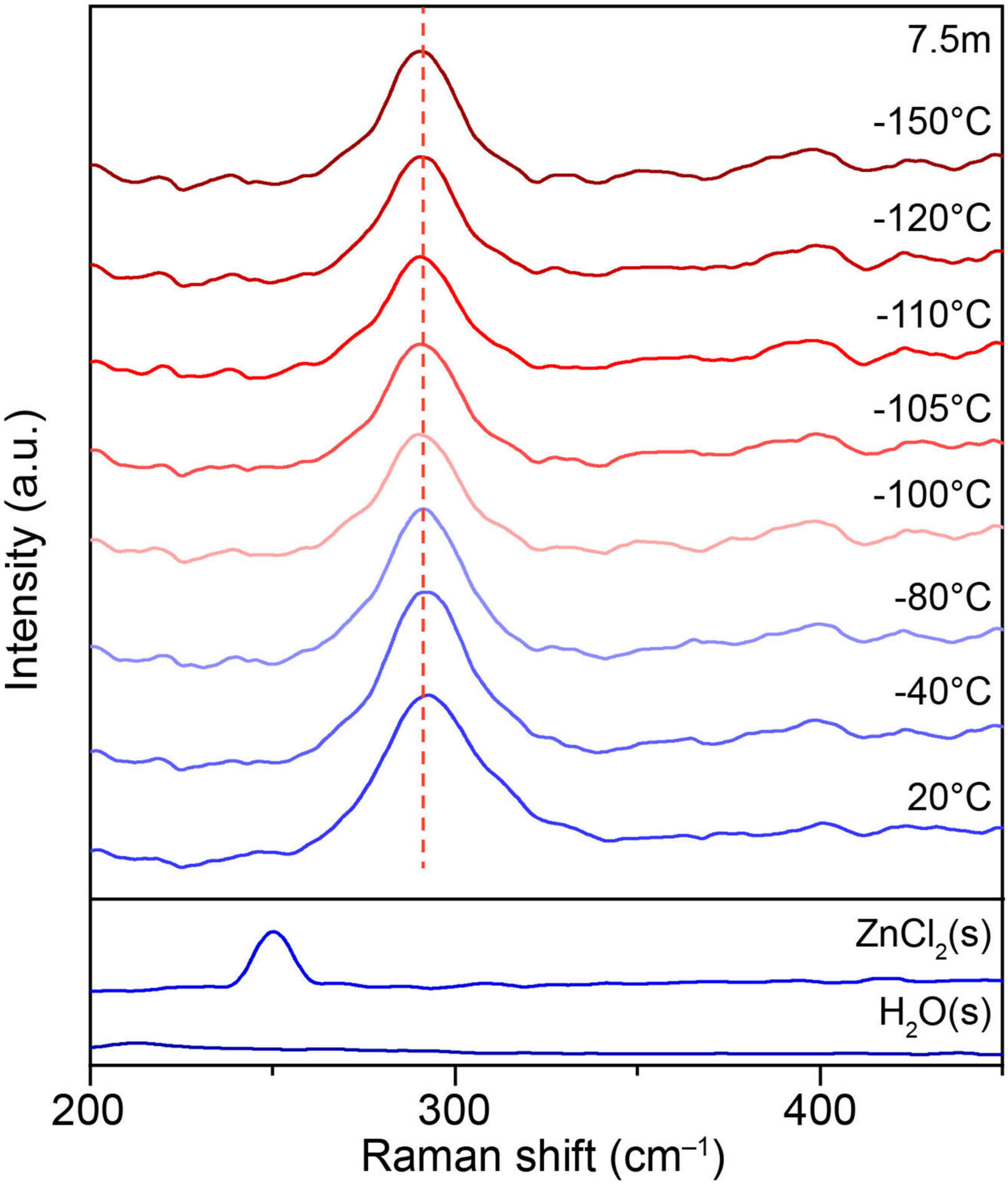

Supplementary Figure 7. Low-temperature Raman spectra of 7.5 m $ZnCl_2$ electrolyte. Undergoing the liquid-glass transition, the structure of electrolyte still remains stable.

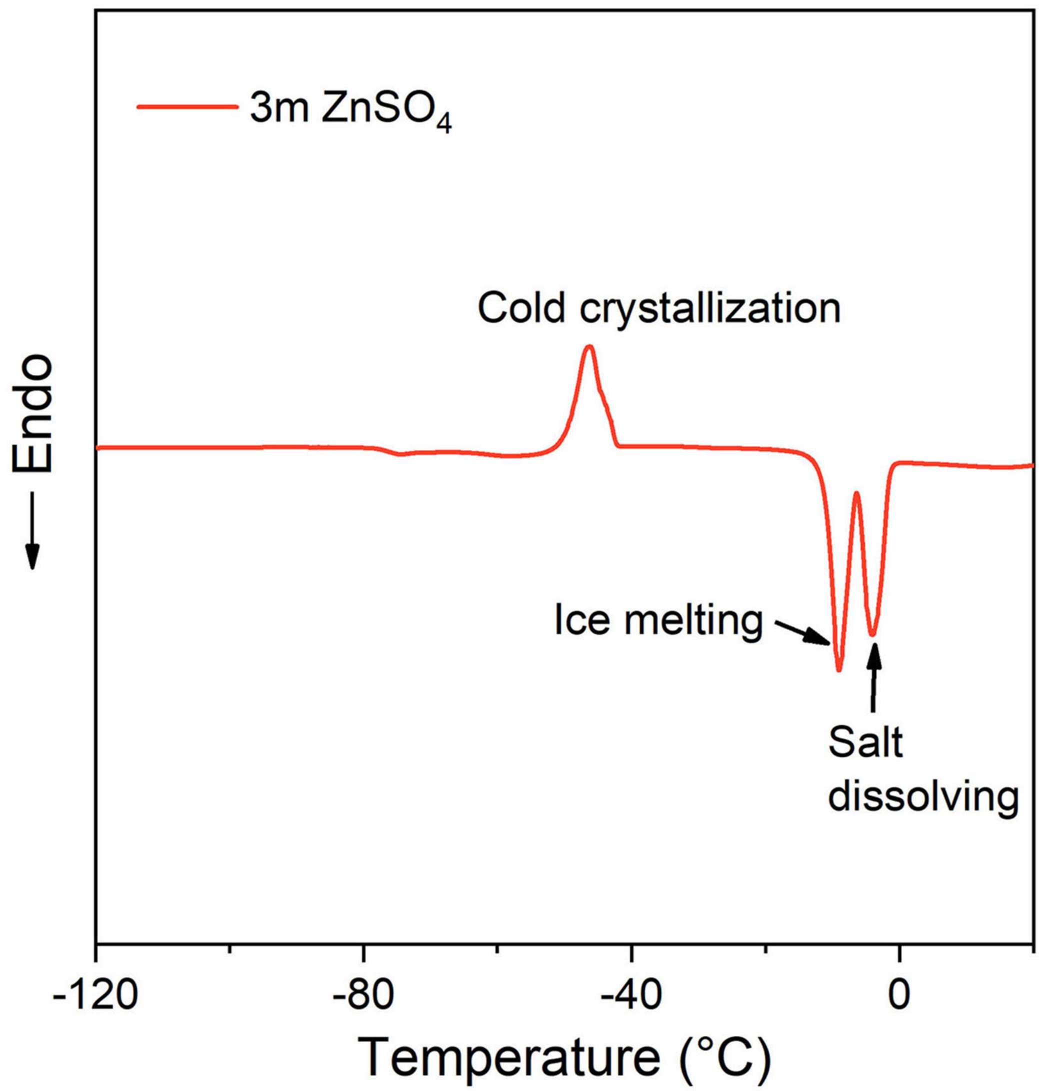

Supplementary Figure 8. DSC curve of 3 m $ZnSO_4$ electrolyte. The peaks in heating process are $ZnSO_4$ cold crystallization, ice melting and salt dissolving respectively. The crystallization in the heat process indicates that the process from amorphous glass transiting to crystal is exothermic, demonstrating that the form of crystal owns lower energy than glass and the transition can occur above –52 °C.

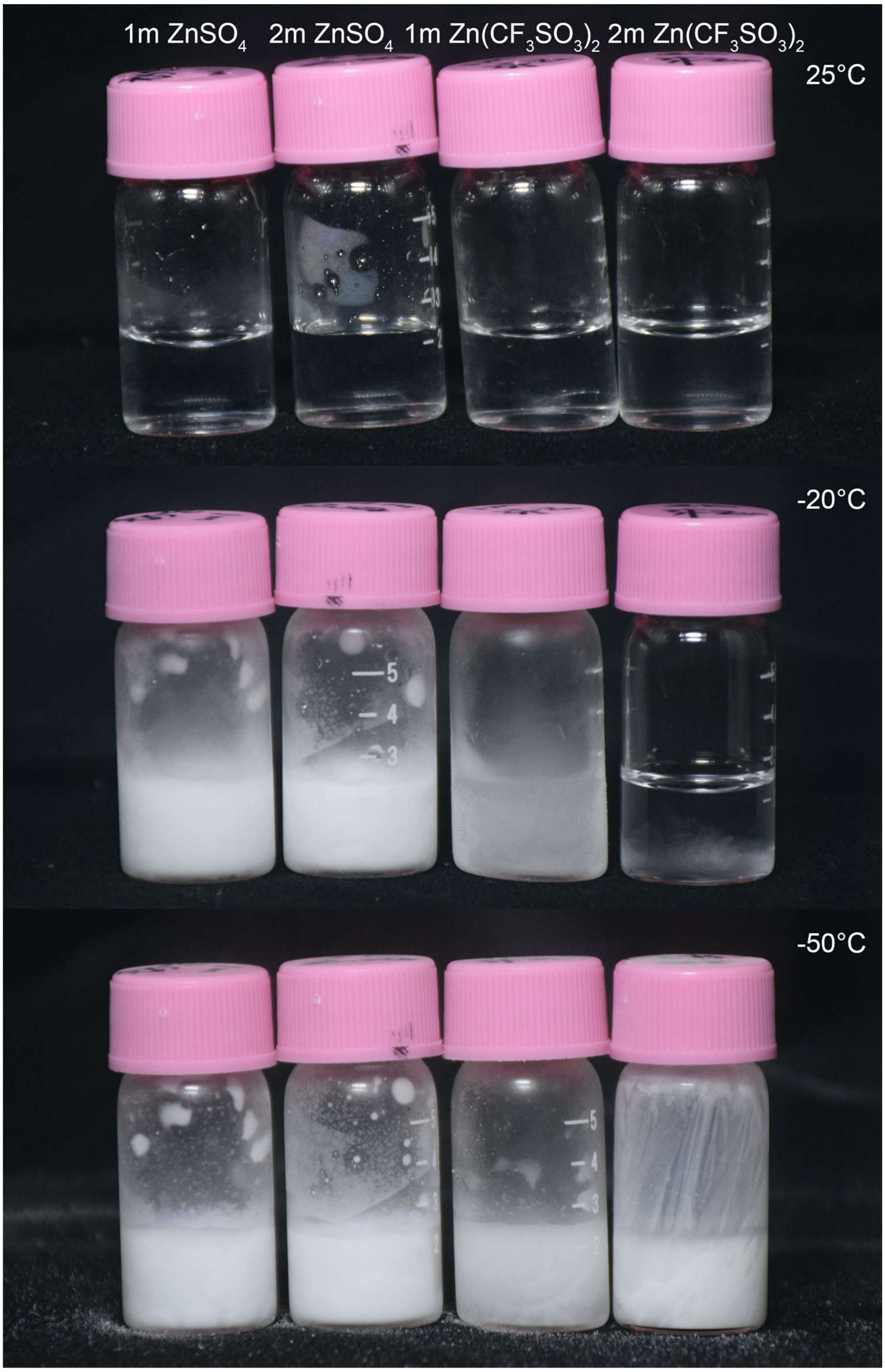

Supplementary Figure 9. Optical photograph of electrolyte at low temperature. 1, 2 m $ZnSO_4$ and 1, 2 m $Zn(CF_3SO_3)_2$ electrolyte at 25, –20 and –50 °C.

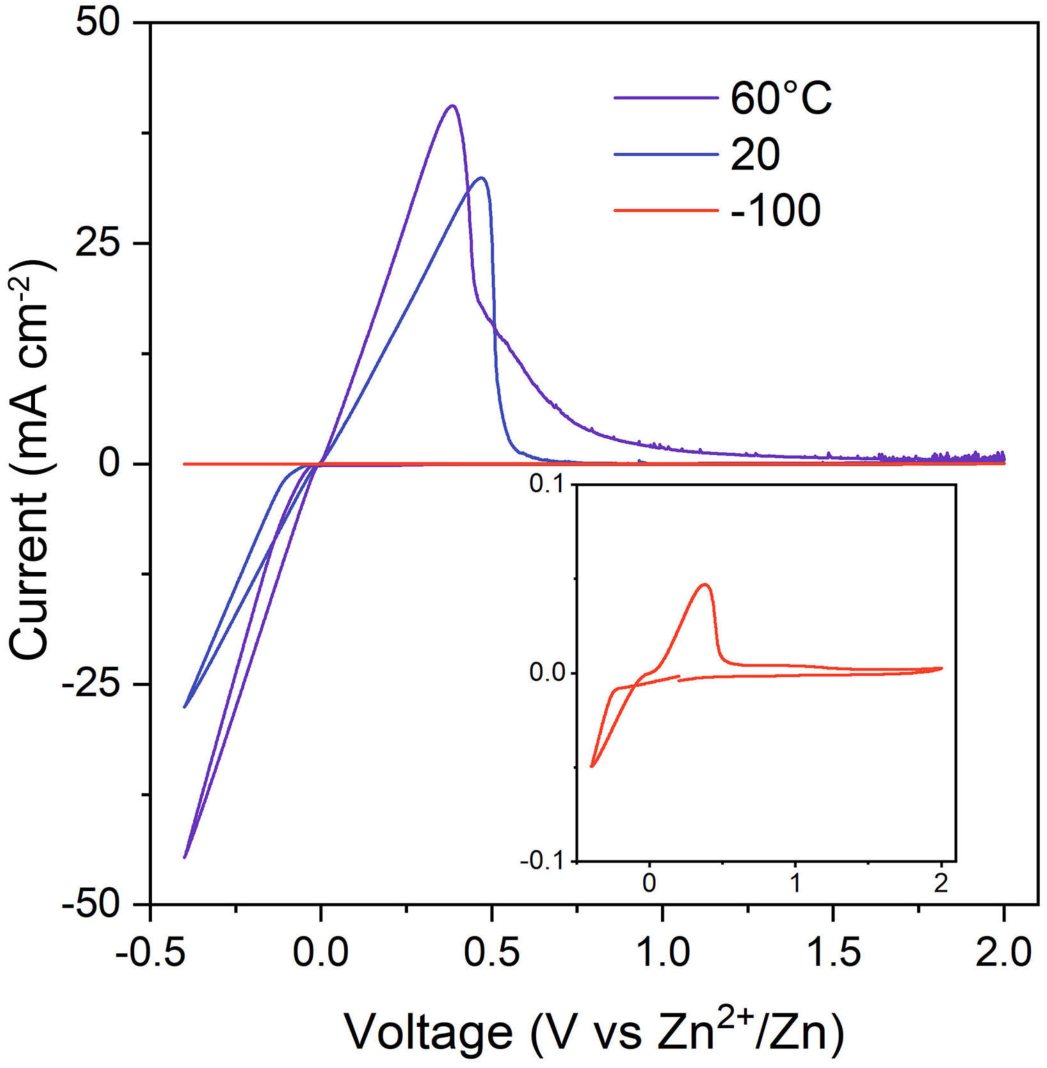

Supplementary Figure 10. CV curves of Zn plating/stripping at 60, 20 and –100 °C. The inset is the magnified CV curve of –100 °C.

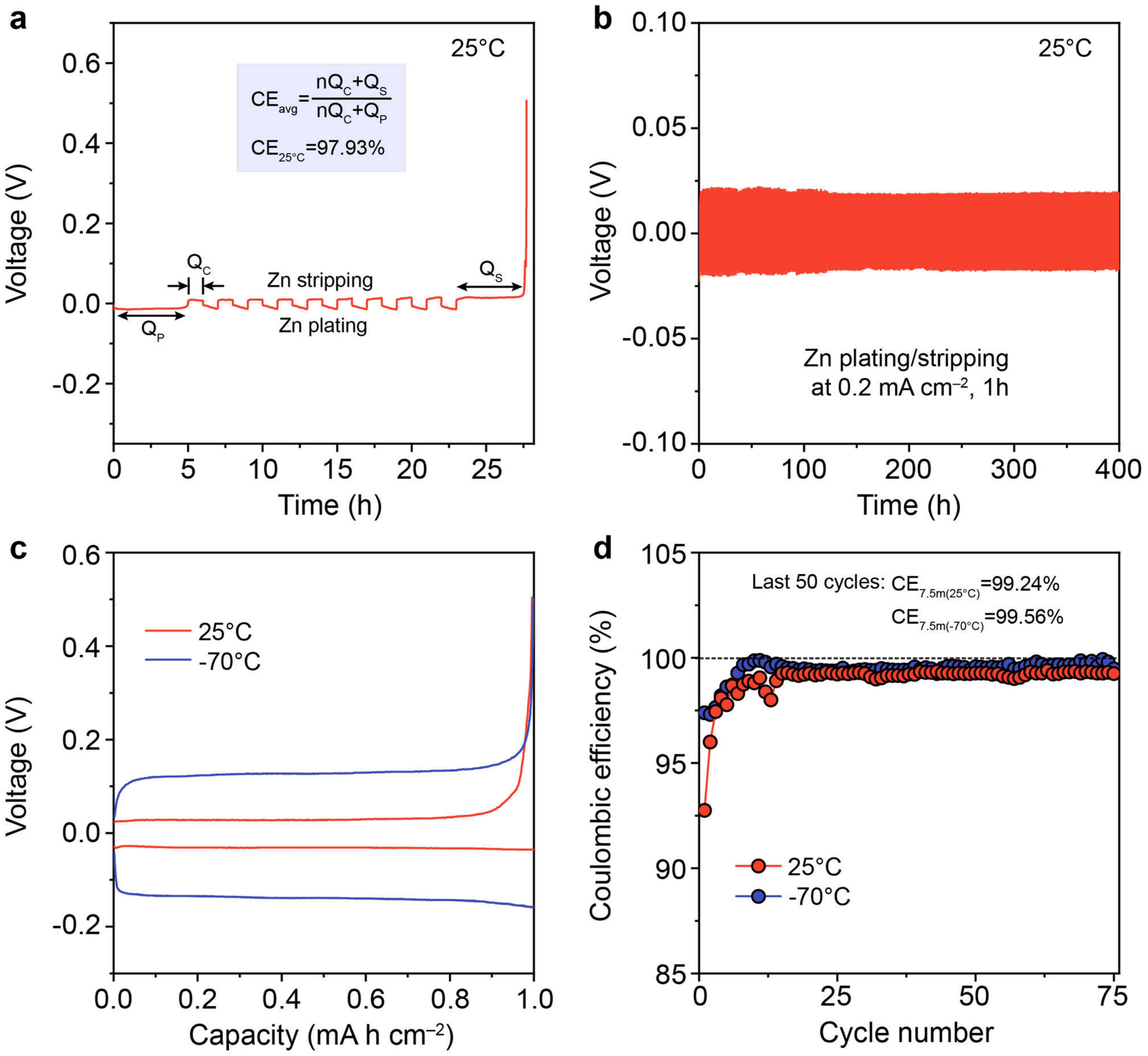

Supplementary Figure 11. The electrochemical performance of Zn metal anode with 1 and 7.5 m $ZnCl_2$ electrolyte. a, The voltage profiles of asymmetric Zn||Cu cell with 7.5 m $ZnCl_2$ electrolyte at 25 °C and 0.2 mA cm^{-2}. b, The cycling performance of symmetric Zn||Zn cell at 25 °C. c, The voltage profiles of asymmetric Zn||Cu cell at 0.2 mA cm^{-2} with 1 and 7.5 m $ZnCl_2$ electrolyte at 25 and –70 °C, respectively. d, The corresponding coulombic efficiencies.

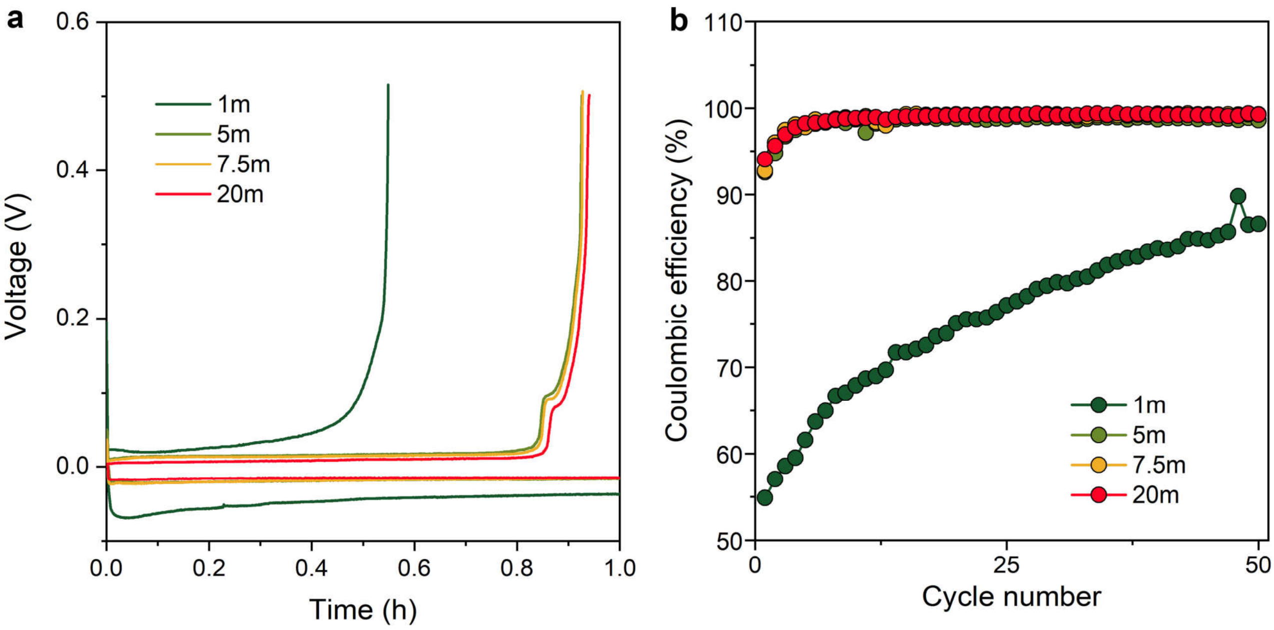

Supplementary Figure 12. The electrochemical performance of Zn metal anode with different C_{ZnCl_2} electrolyte at 25 °C. a, The first cycle of Zn plating/stripping at the current density of 0.2 mA cm^{-2}, b, The corresponding coulombic efficiencies of Zn||Cu asymmetric cells.

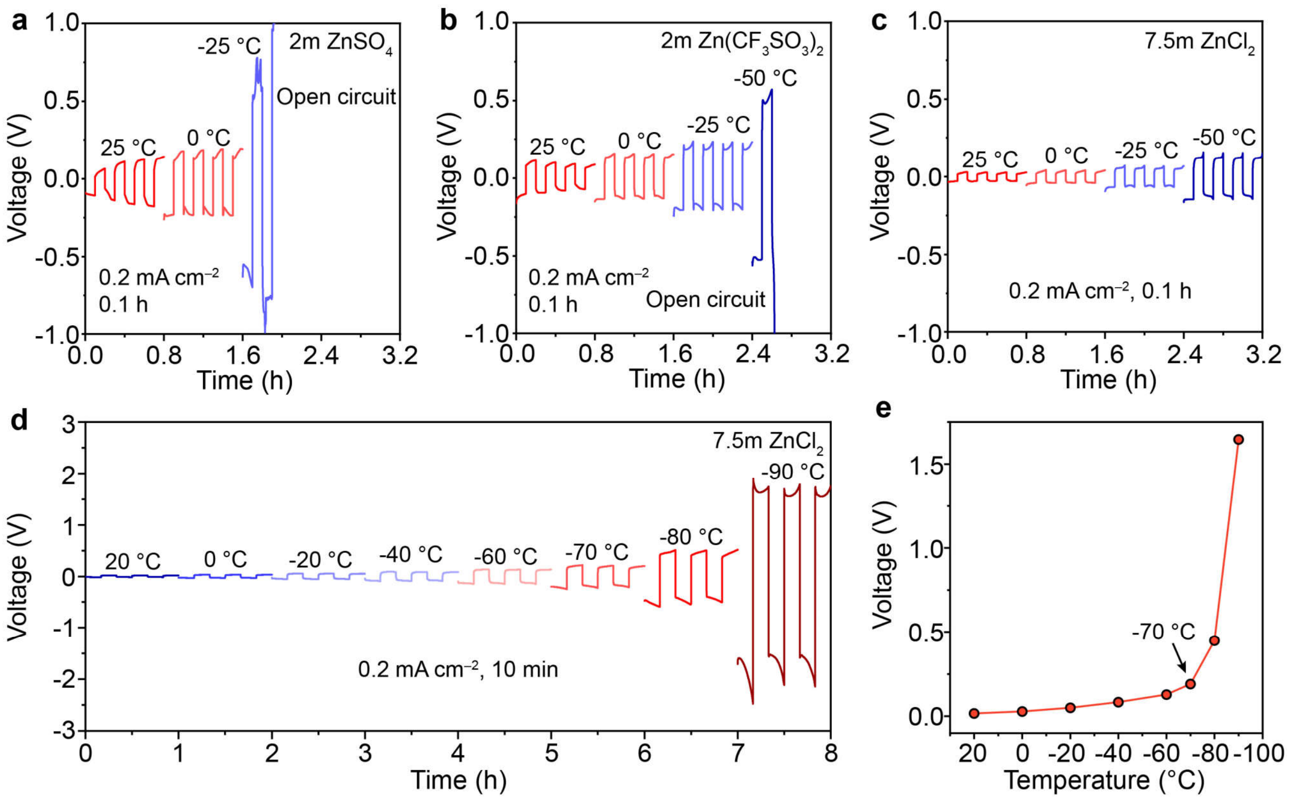

Supplementary Figure 13. The voltage profiles of the symmetric Zn||Zn cells based on a, 2 m $ZnSO_4$, b, 2 m $Zn(CF_3SO_3)_2$ c, 7.5 m $ZnCl_2$ electrolyte (LTE) at 25, 0, –25 and –50 °C with the current density of 0.2 mA cm^{-2}. d, The voltage profiles of the symmetric Zn|LTE|Zn cell testing at various temperature. e, The corresponding overpotential with temperature.

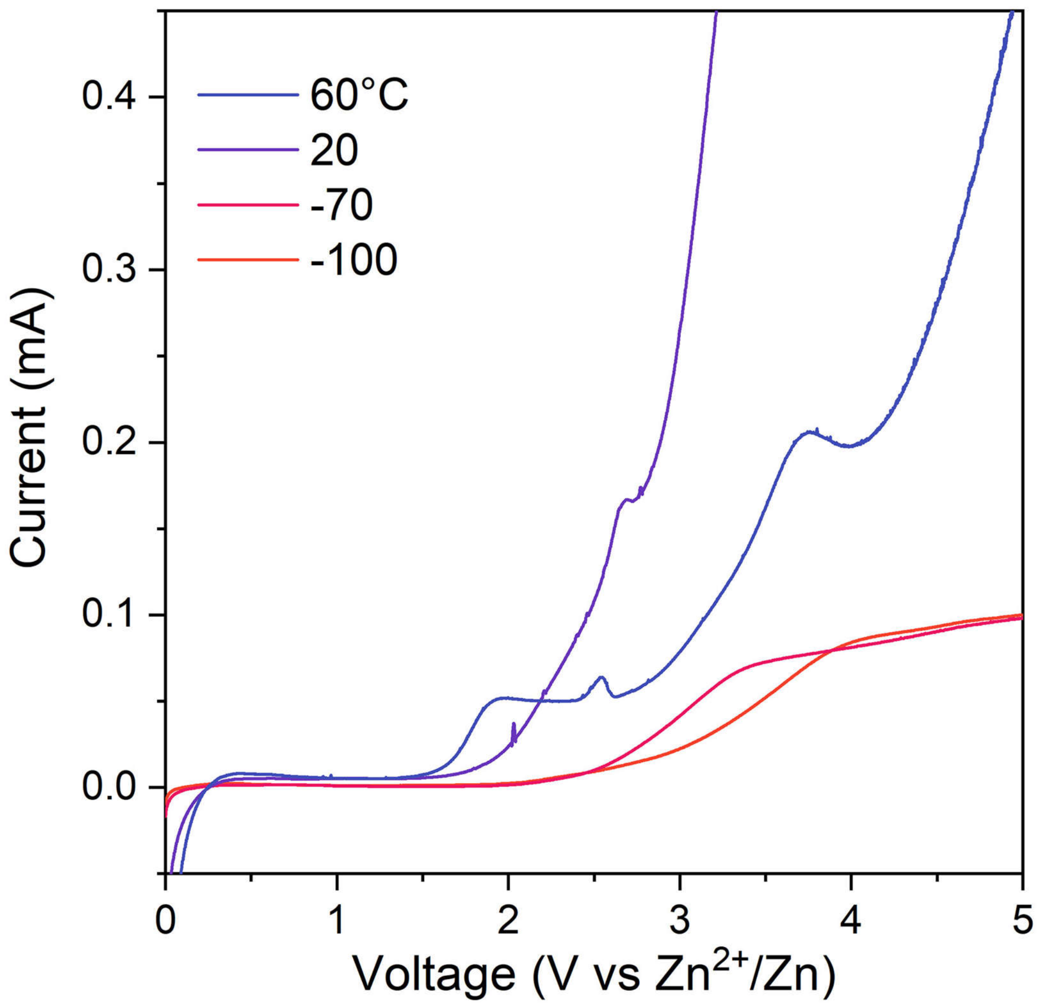

Supplementary Figure 14. Electrochemical windows of 7.5 m $ZnCl_2$ electrolyte at 60, 20, –70 and –100 °C. The corresponding electrochemical windows are 1.6, 1.8, 2.3 and 2.3 V, respectively.

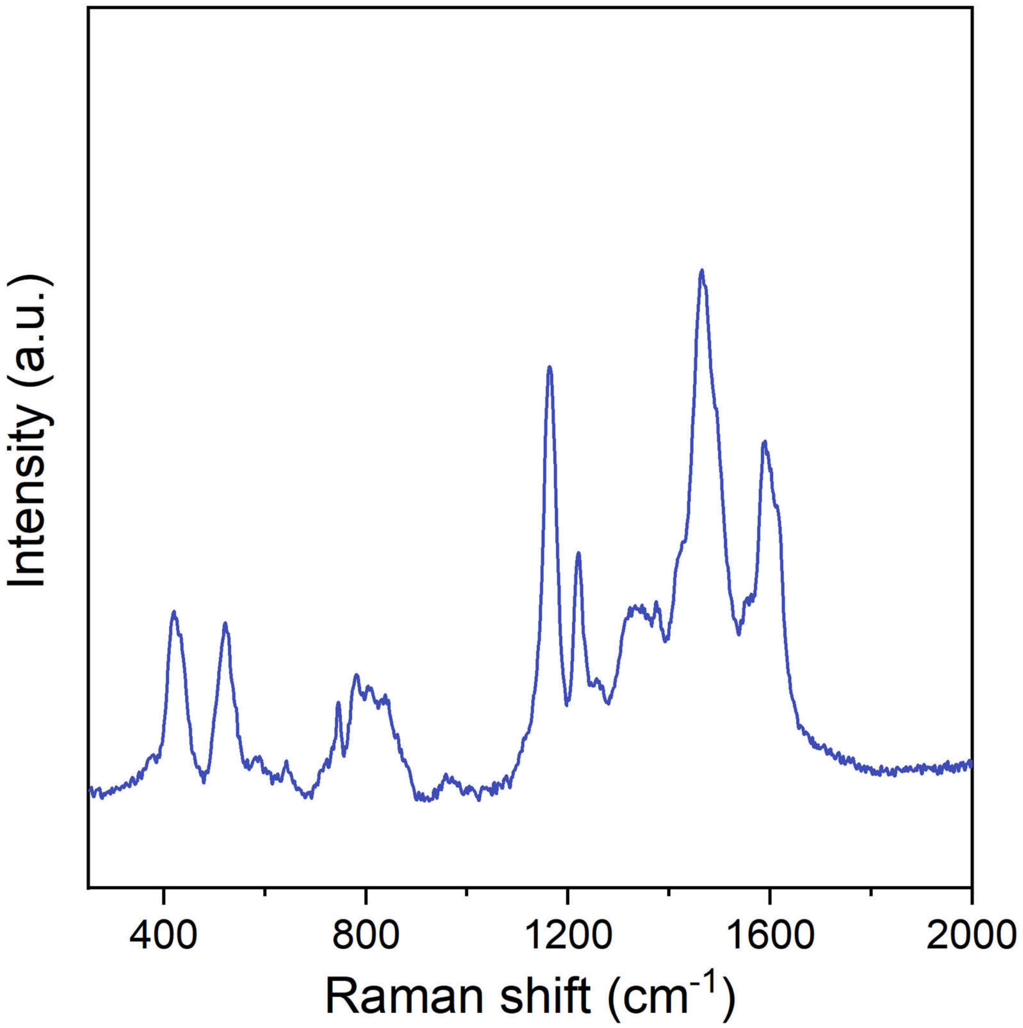

Supplementary Figure 15. Raman spectrum of obtained PANI powder. The peaks at 419.6/520.9 cm^{-1} (C–C out-of-plane deformation), 745.5/783.1 cm^{-1} (out of-plane C–H bending of quinone ring), 804.3/837.1 cm^{-1} (out of-plane C–H bending of benzene ring), 1163.0/1220.8 cm^{-1} (C–H in-plane deformation vibration of quinone ring), 1464.8 cm^{-1} (C=N, C=C stretching vibration) and 1590.1 cm^{-1} (quinone ring structures) demonstrates the highly purified PANI.

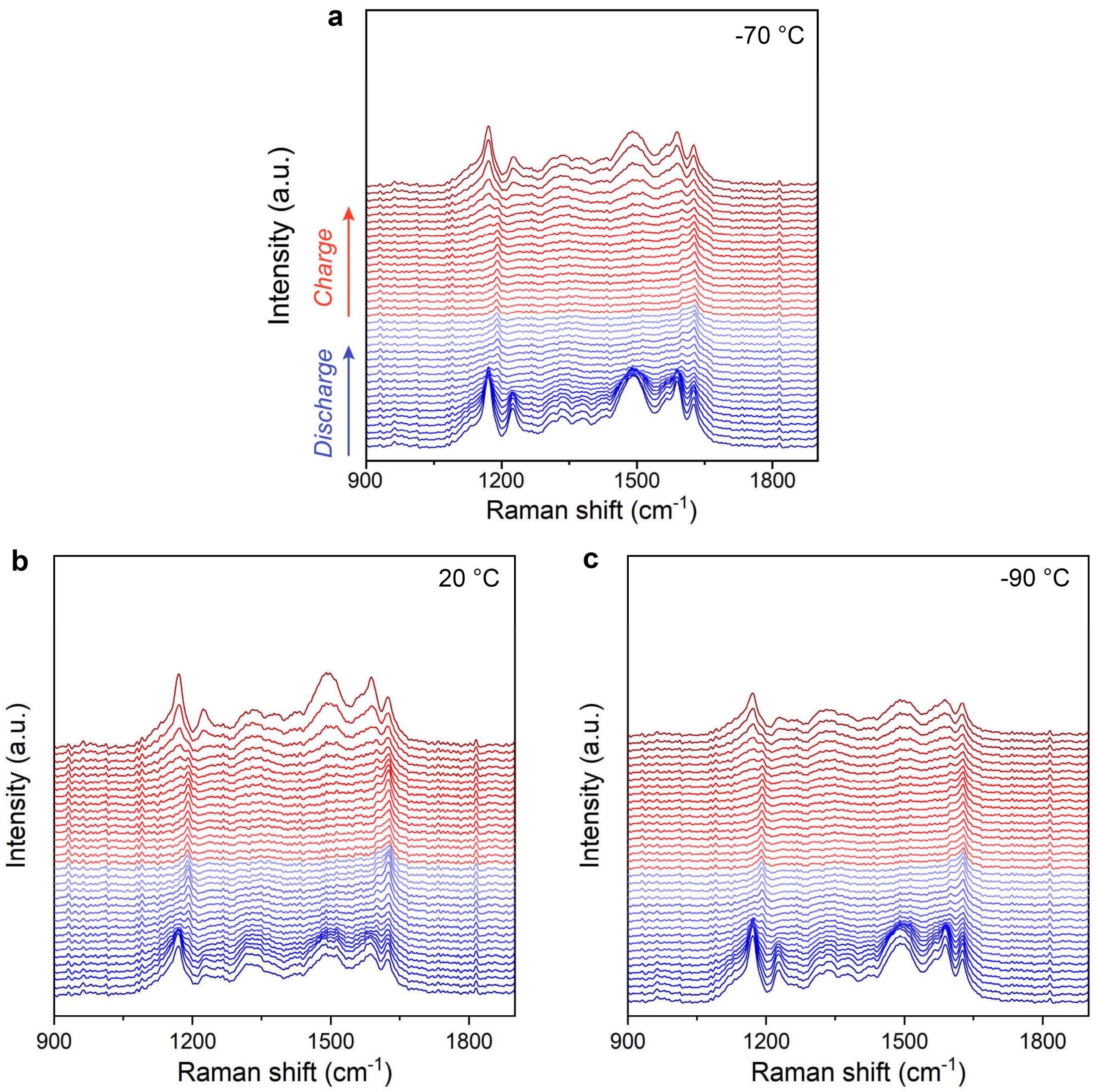

Supplementary Figure 16. In situ Raman spectrums of PANI in the electrochemical redox processes at the different temperature. a, –70 °C. b, 20 °C. c, –90 °C.

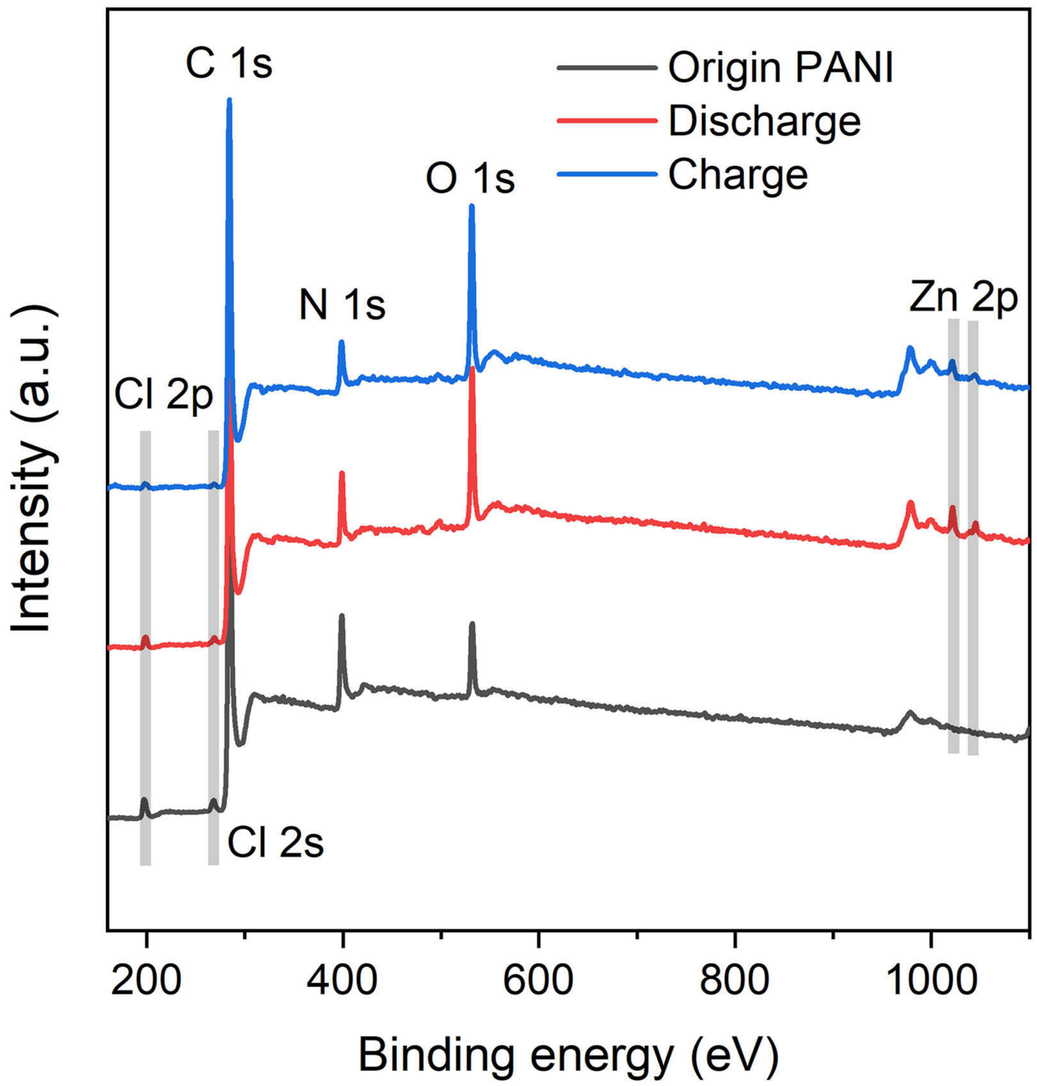

Supplementary Figure 17. XPS profiles of PANI electrode in the original, discharge and charge states. The PANI samples in the discharge and charge state contain both Zn and Cl elements.

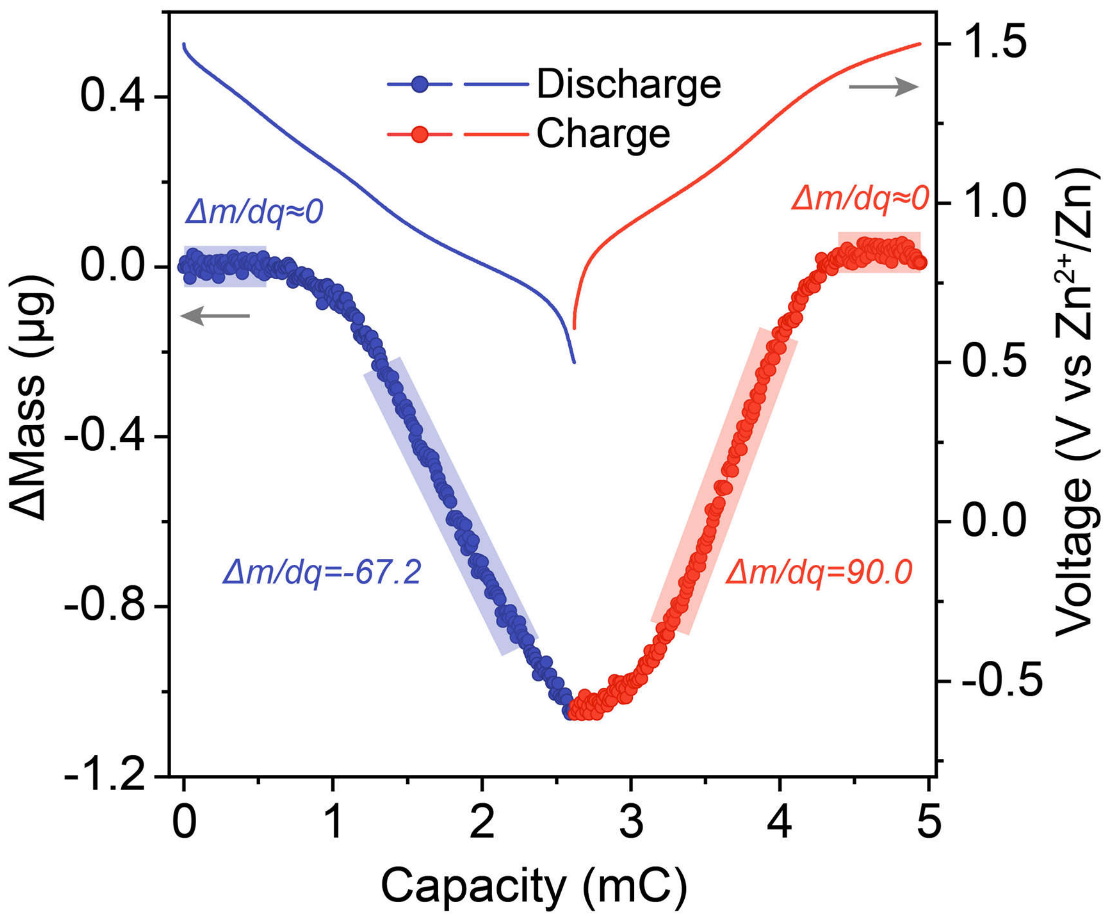

Supplementary Figure 18. In situ EQCM profiles of PANI electrode during discharge/charge process at the current of 10 μA.

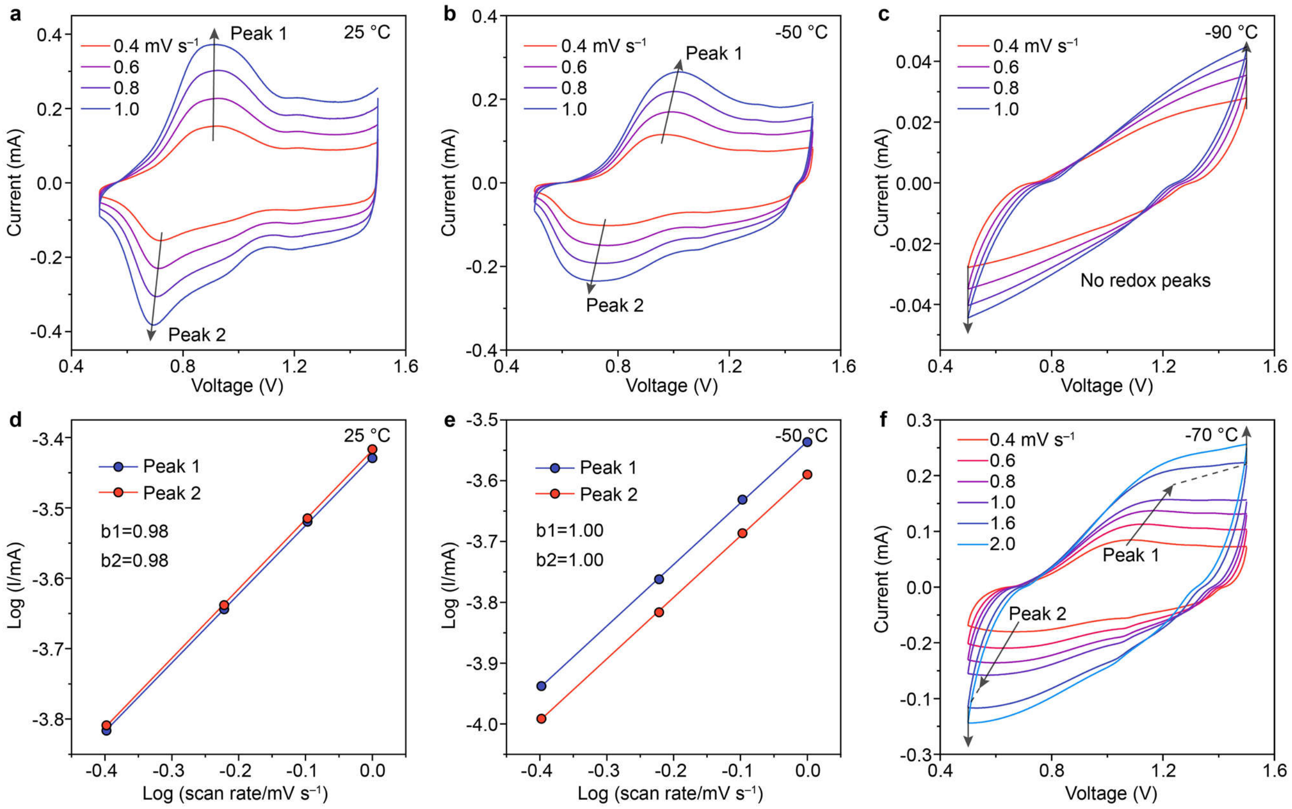

Supplementary Figure 19. a-c, CV curves of PANI|LTE|Zn batteries at 25, –50 and –90 °C. d-e, The fitted line of peak currents and scan rates according to the equation $log(i)=log(a)+b\times log(v)$. The b values are close to 1, representing the domination of the pseudo-capacitance behavior in the charge/discharge process, while the b values are close to 0.5, representing that the reaction is controlled by ionic diffusion. With the temperature decreasing, the b values are increased, indicating that the electrochemical process at low temperature is closer to pseudo-capacitance behavior than that at room temperature. The peak current was calibrated based on the ratio of capacities. f, CV curves of Zn||PANI batteries at –70 °C. With the scan rate increasing, the gaps of the peaks in redox process are also increased. At 1.6 and 2.0 mV s^{-1}, the redox peaks disappear, which is similar with that at –90 °C and even at low scan rate. It demonstrates that the both high current density and low temperature can result in the charge-discharge curves without plateau.

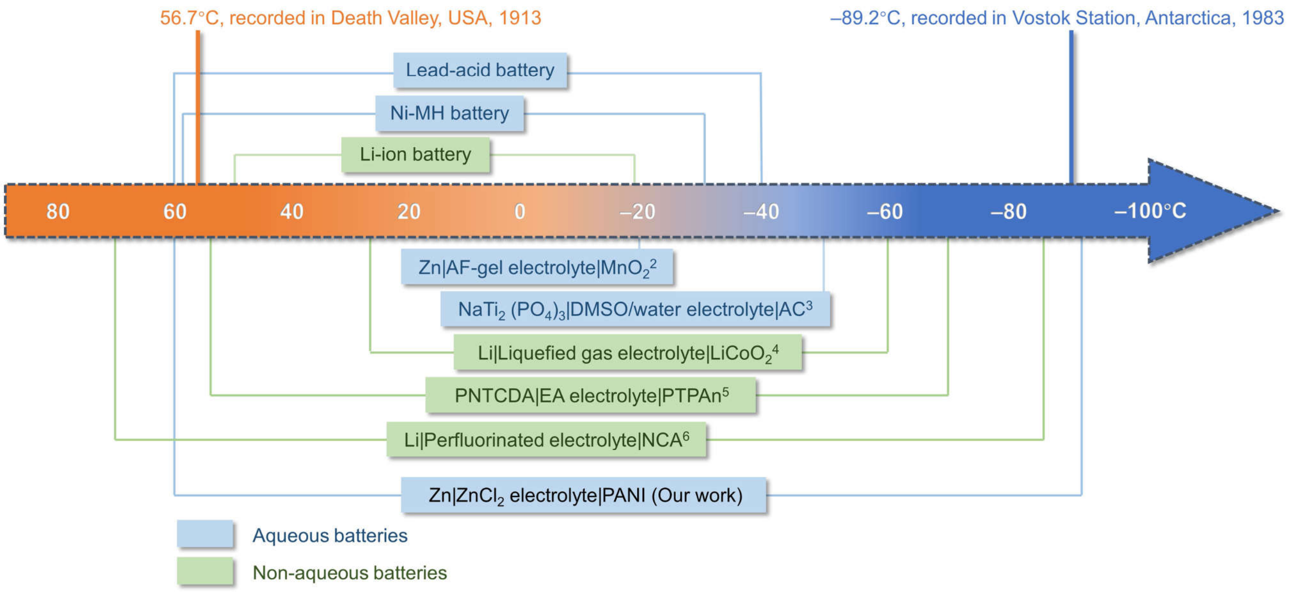

Supplementary Figure 20. The operation temperature windows of the present batteries.

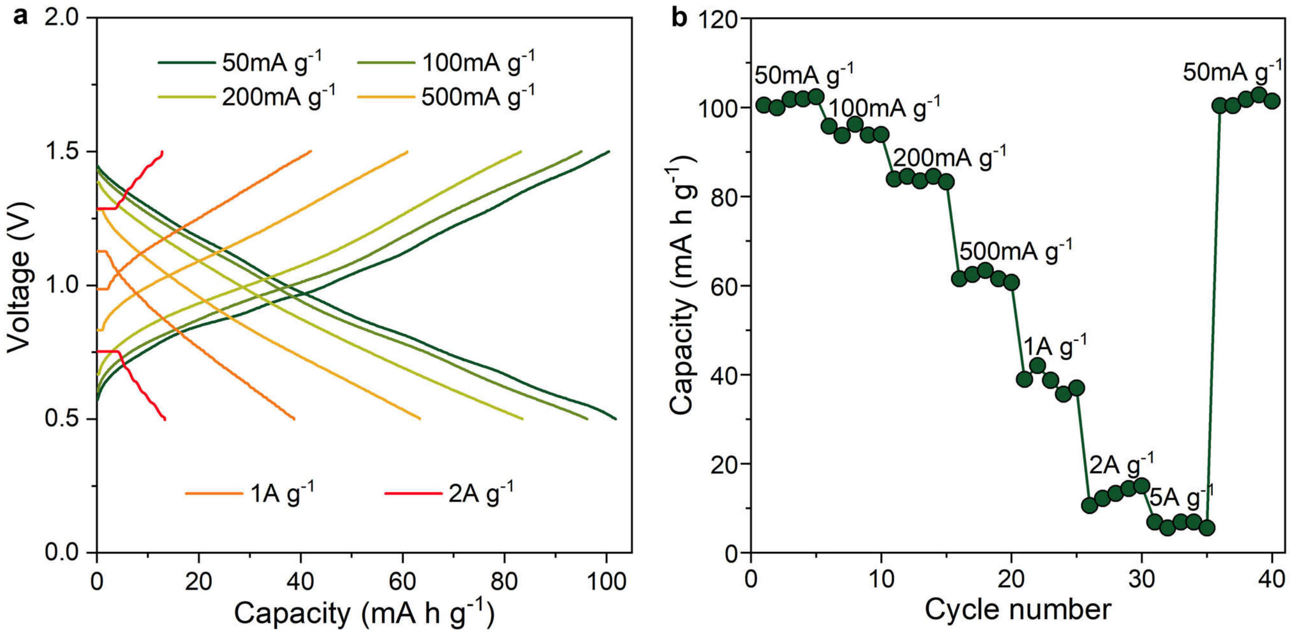

Supplementary Figure 21. The rate performance of PANI|LTE|Zn batteries at –70 °C. a, The charge-discharge curves at different current densities. b, The corresponding discharge capacities.

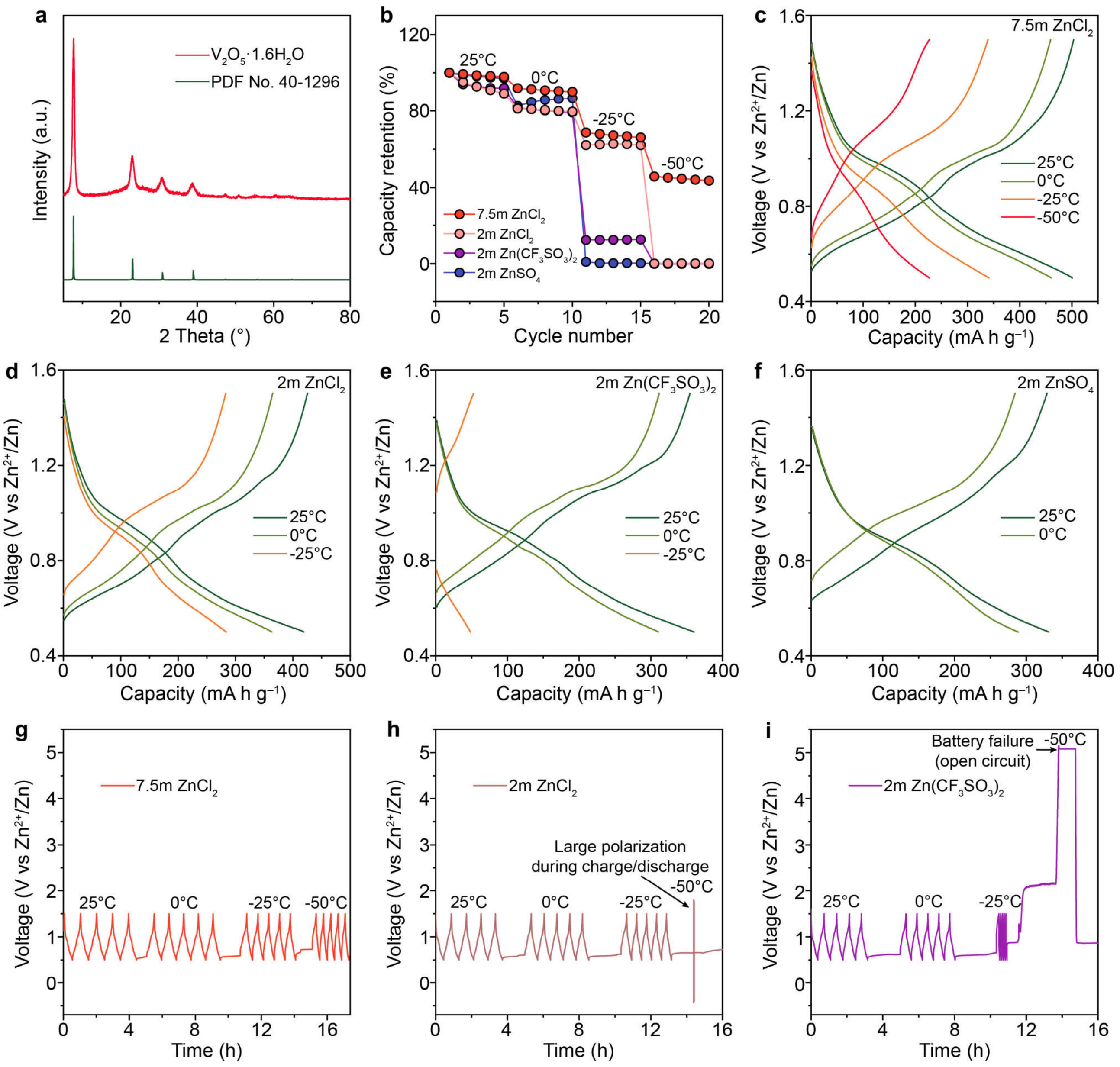

Supplementary Figure 22. The XRD profiles and electrochemical performance of $V_2O_5{\cdot}1.6H_2O$. a, XRD of $V_2O_5{\cdot}1.6H_2O$. b, Electrochemical performance of the batteries using $V_2O_5{\cdot}1.6H_2O$ cathode and 7.5 m $ZnCl_2$, 2 m $ZnCl_2$, 2 m $Zn(CF_3SO_3)_2$ and 2 m $ZnSO_4$ electrolyte at 25, 0, –25, –50 °C, respectively. c-f, The corresponding voltage-capacity curves at the current of 1 A g^{-1}. g-h, The corresponding voltage-time curves. The 7.5 m $ZnCl_2$, 2 m $ZnCl_2$, 2 m $Zn(CF_3SO_3)_2$ electrolyte are unfrozen, partially frozen and fully frozen states at –50 °C, resulting in the normal operation, large polarization during charge/discharge and battery failure (open circuit).

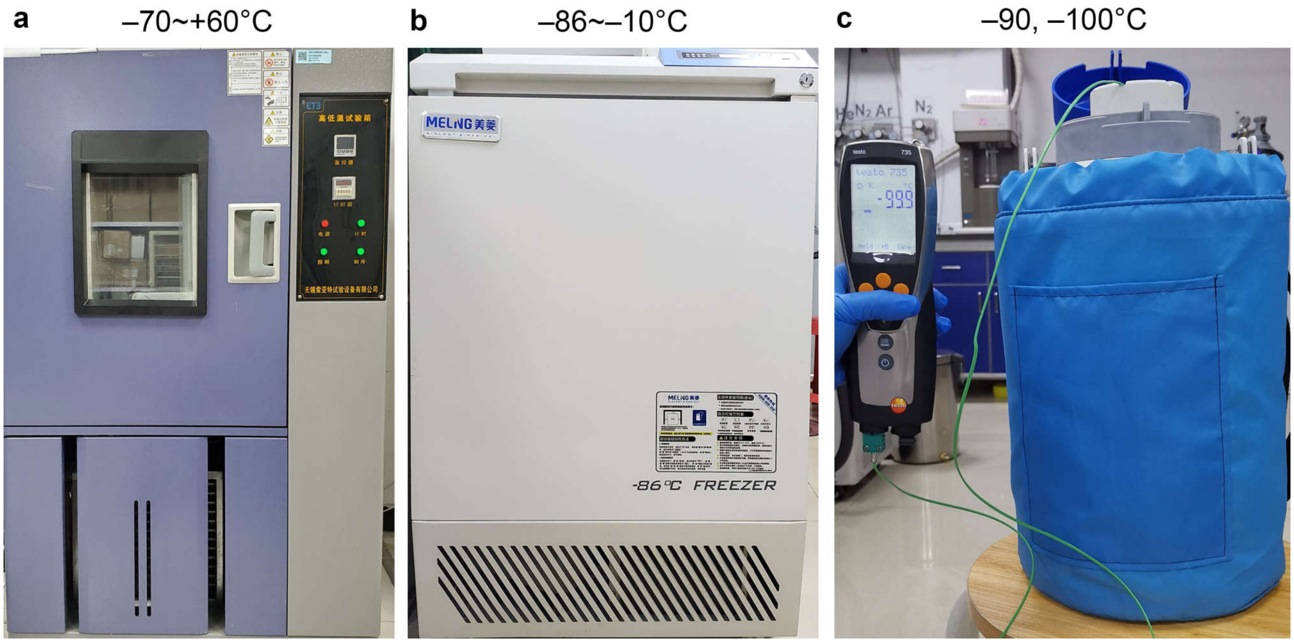

Supplementary Figure 23. The three types of temperature control devices and the corresponding temperature control range. a, Suoyate WuXi (–70~60 °C). b, Meiling refrigerator DW-HW50 (–86~–10 °C). c, Melting solid n-Heptane or methanol in an insulation container (–90, –100 °C).

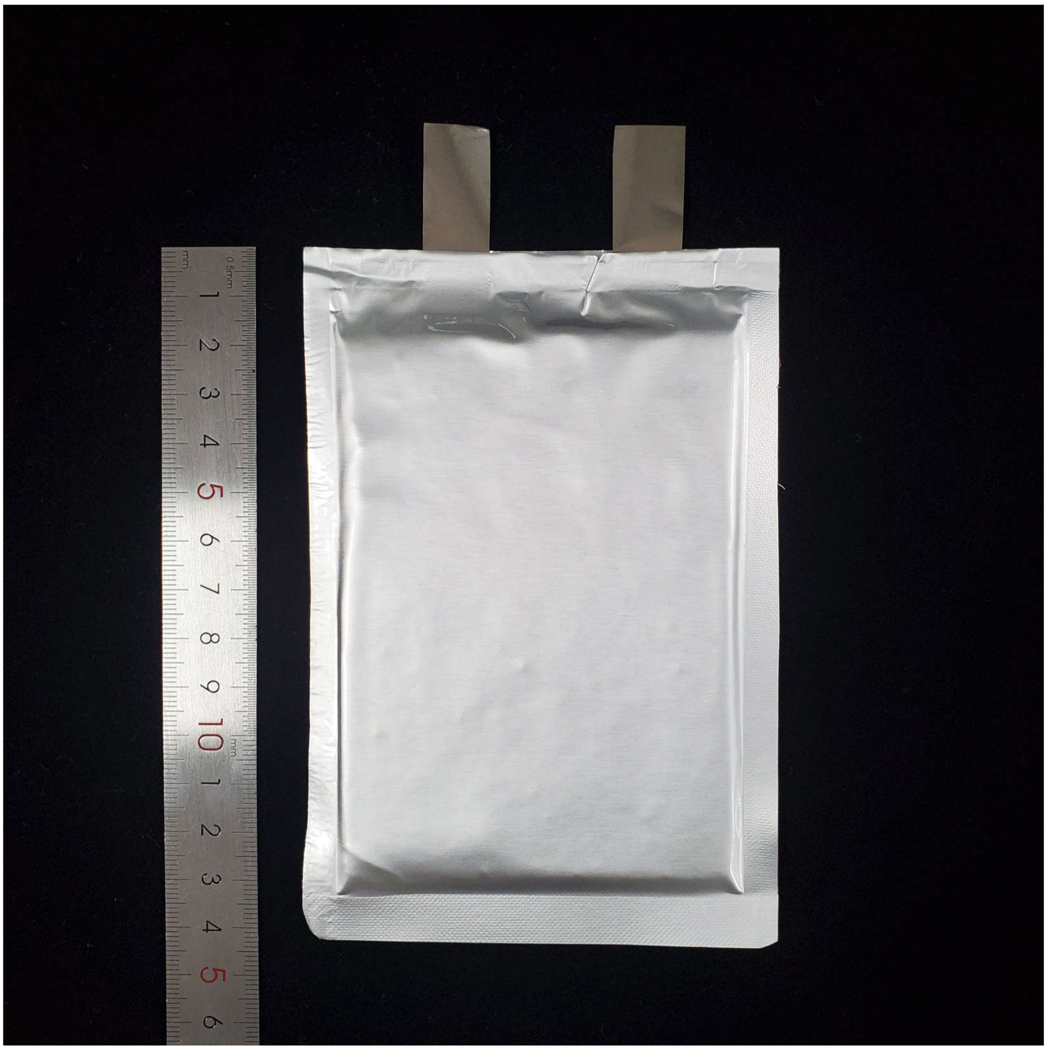

Supplementary Figure 24. The optical photograph of pouch cell.

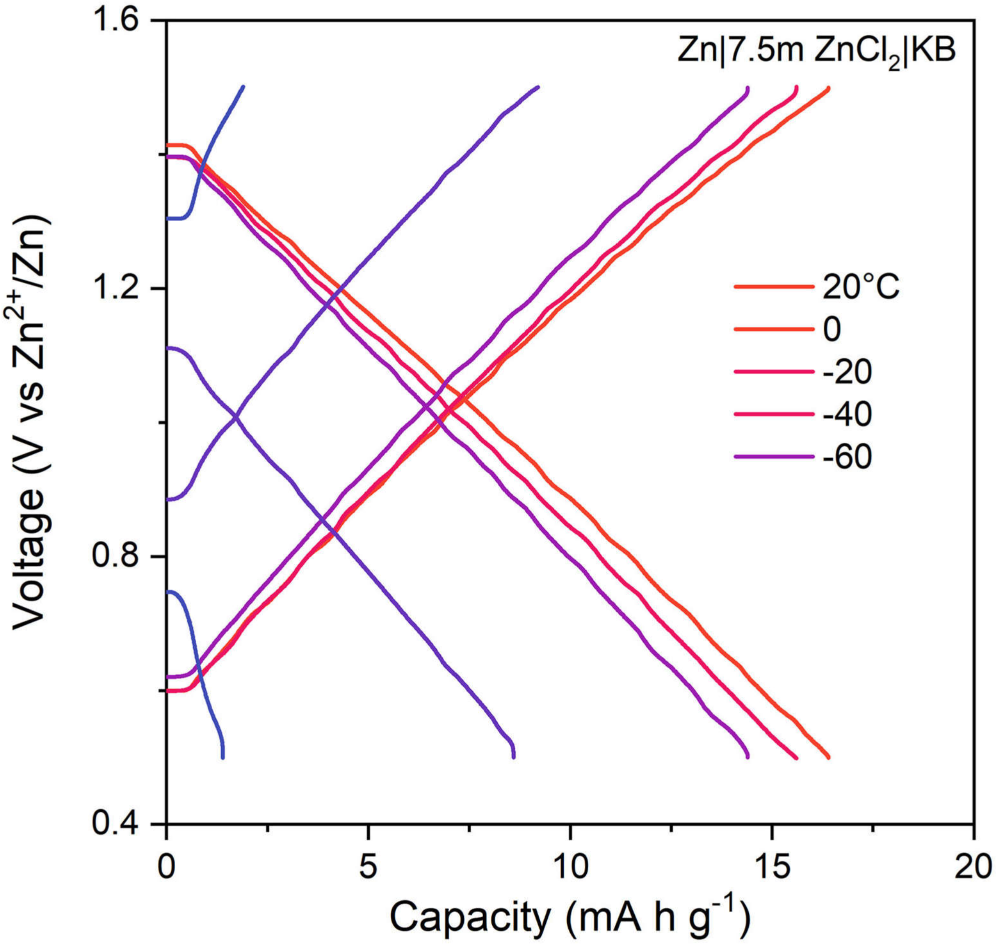

Supplementary Figure 25. The discharge-charge curves of Zn|7.5 m $ZnCl_2$|KB battery at different temperature.

Supplementary Table 1. Solid-liquid transition temperature of $ZnCl_2$ electrolyte from DSC tests. The major transition temperature is highlighted in bold.

$ZnCl_2$ concentration (mol kg^{-1})	Glass-liquid transition temperature (°C)	Ice melting temperature (°C)	Salt dissolving temperature (°C)
0	–	**0.0**	–
1	–109.3	**–12.6**	–
3	–109.8	**–39.5**	–
5	–109.7	**–46.0**	–
7.5	**–114.2**	–	–
10	**–105.8**	–	–
15	**–60.2**	–	–
20	**–46.7**	–	–
30	–67.1	–	**1.2**

Supplementary Table 2. The ionic conductivities of 1, 5, 7.5, 10 and 30 m $ZnCl_2$ and 2 m $ZnSO_4$ and 2 m $Zn(CF_3SO_3)_2$ electrolyte.

Temperature (°C)	Ionic conductivities (mS cm^{-1})						
	1 m $ZnCl_2$	5 m $ZnCl_2$	7.5 m $ZnCl_2$	10 m $ZnCl_2$	30 m $ZnCl_2$	2 m $ZnSO_4$	2 m $Zn(CF_3SO_3)_2$
60	109.3	145.6	132.4	103.1	35.9	93.5	112
40	96.4	117.6	103.6	74.0	16.7	71.5	88.4
20	77.7	89.2	73.3	56.9	4.47	52.4	60.9
0	54.7	58.0	44.8	30.8	5.18E-1	38.839	41.0
–20	8.51	31.4	21.2	12.8	9.83E-2	4.80E-5	16.1
–40	2.05	8.92	8.68	3.41	4.21E-4	6.41E-09	9.19E-1
–60	4.40E-1	8.42E-1	1.79	1.11	1.03E-7	2.52E-11	3.79E-08
–80	9.27E-3	3.08E-2	4.24E-1	1.85E-1	–	–	–
–100	2.14E-4	3.50E-4	1.70E-2	1.42E-3	–	–	–

Supplementary Table 3. The activation energy of the electrolyte at different temperature range.

Electrolyte	Activation energy (eV)	
	Stage I	Stage II
1 m $ZnCl_2$	0.091 (60~0°C)	0.511 (–20~–100°C)
5 m $ZnCl_2$	0.181 (60~–40°C)	0.620 (–60~–100°C)
7.5 m $ZnCl_2$	0.184 (60~–40°C)	0.374 (–60~–100°C)
10 m $ZnCl_2$	0.225 (60~–40°C)	0.536 (–60~–100°C)
30 m $ZnCl_2$	0.556 (60~0°C)	1.610 (–20~–60°C)
2 m $ZnSO_4$	0.115 (60~0°C)	3.067 (0~–40°C)
2 m $Zn(CF_3SO_3)_2$	0.172 (60~–40°C)	3.641 (–40~–60°C)

Supplementary Table 4. Cell parameters of the Zn||PANI pouch cell at the 1.0 Ah level.

	Parameter	Value
PANI cathode	Discharge capacity	151 mA h g^{-1} at 1 A g^{-1}
	Active material loading	60%
	Area weight	17.3 mg cm^{-2}
	Area capacity	2.6 mA h cm^{-2}
	Number of layers	5
Binder	Type	PTFE
Ti mesh current collector	Area weight	12.8 mg cm^{-2}
Zn metal anode	Specific capacity	820 mA h g^{-1}
	Zn thickness	20 μm
	Area capacity	11.7 mA h cm^{-2}
	N/P ratio	2.2
Electrolyte	Concentration	7.5 m
	E/C ratio	4.0 g Ah^{-1}
Separator	Type	Celgard 3501
	Thickness	25 μm
	Area weight	12.9 g m^{-2}
Package foil	Thickness	115 μm
Cell	Average voltage	0.91 V
	Capacity	1.15 Ah
	Cell energy based on the mass of active materials	97.9 Wh kg^{-1} at 20 °C<br>42.6 Wh kg^{-1} at –70 °C
	Cell energy based on the total mass of cell	38.9 Wh kg^{-1} at 20 °C<br>16.9 Wh kg^{-1} at –70 °C

Supplementary Table 5. MD parameters of $ZnCl_2$[1].

Atoms	*q/e*	*σ/Å*	*ε/kJ mol⁻¹*
Zn^{2+}	2.00	1.95998	5.23000e–02
Cl^{-}	−1.00	4.40104	4.18400e–01

Supplementary Note 1. The calculation of the change of H-bond number in the water/ice phase transformation.

The H-bond formation energy (E_{HB}) in ice is obtained when transforming one H-bound O–H···O group in ice into one free O-H group and can be expressed as:

$$E_{HB} \approx -23 \text{ kJ mol}^{-1}$$

On the other hand, the heat of fusion of ice (ΔH_f) is equal to:

$$\Delta H_f \approx 6 \text{ kJ mol}^{-1}$$

Supposing that the broken H-bond number per water molecules during the transformation from ice to water is C. Considering that the H_2O is both H-bond donor and acceptor. Thus, the C is twice as the broken H-bond number:

$$C = 2 \times E_{HB} / \Delta H_f$$

As a result, the C is equal to 0.52.

Supplementary Note 2. The Raman spectroscopy analysis of PANI cathode.

The peaks at 1171.0, 1225.7 cm^{-1} represents the in-plane deformation vibration of C-H in quinone ring. C=C and C=N stretching vibration display the peak of 1490.7 cm^{-1}. The peaks located at 1589.0 and 1625.4 cm^{-1} are regard as quinone and benzene ring structures respectively. Fig. 5a shows the reversible Raman signal changes of PANI cathode during the discharge-charge processes at –70 °C. During the discharge process, the peaks of quinone structure disappear, and the peaks representing the benzene ring structures emerge at 1189.3 and 1628.5 cm^{-1}. With charging, the peaks recover.

Supplementary Note 3. The ions compensation mechanism of PANI cathode.

With the internal structure transforming, cathode maintains charge balance by ions adsorption/desorption. During discharging, the cathode is reduced and will adsorb cations and/or desorb anions. During charging, the cathode is oxidized and will adsorb anions and/or desorb cations. As a result, the electrode adsorbs cations/anions finally at the state of full discharge/charge. However, the coexistence of Zn and Cl at both full discharge and charge states in X-ray photoelectron spectroscopy (XPS) profiles indicates that the ions compensation mechanism of PANI cathode in $ZnCl_2$ electrolyte is not Zn^{2+} adsorption/desorption and Cl^- desorption/adsorption in discharge/charge process, otherwise, the XPS profiles should show that no Zn signal in the charge state and no Cl signal in the discharge state (Supplementary Figure 17). The adsorbed ions contain both Zn and Cl. Thus, we infer that Zn-Cl complex involves in the adsorption/desorption, instead of itself dissociating and then adsorbing.

To reveal the ion species involving in the compensation mechanism, in situ electrochemical quartz crystal microbalance (EQCM) was utilized (Supplementary Figure 18). In EQCM profiles, there are two regions in the discharge and charge processes, respectively. In the high voltage region of near 1.5 V, the ratio of mass change to charge ($\Delta m/dq$) is almost 0, indicating the light ions (such as H^+) adsorption/desorption occurs in this region. In low voltage region, the huge $\Delta m/dq$ of –67.2 and 90.0 g mol^{-1} of e^- also implies that it is not induced by simple ions, such as Zn^{2+} and Cl^-, which cause $\Delta m/dq$ equal to 32.7 and 35.5 g mol^{-1} of e^-, respectively.

With discharging, the possible reaction mechanism can be concluded as cations adsorption, and/or anions desorption. In EQCM profiles, the mass of electrode decreases with the discharge of depth, implying that there is no cations adsorption solely, which will increase the mass of electrode. Thus, the decrease of electrode mass can only be caused by that the heavy anions desorption and light cations adsorption occurs simultaneously with discharging. The $\Delta m/dq$ based on $Zn(H_2O)_2Cl_4^{2-}$ desorption, $ZnCl^+$ (or H^+) adsorption is calculated as −21.8 (or −121.7) g mol^{-1} of e^-, and the specific $\Delta m/dq$ depends on the ratio of the two reactions. The corresponding reduction processes can be concluded as followed:

Reaction involving H^+:

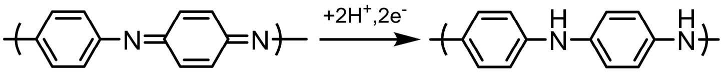

$\Delta m/dq = +1$ g mol^{-1} of e^-

Reaction involving anion:

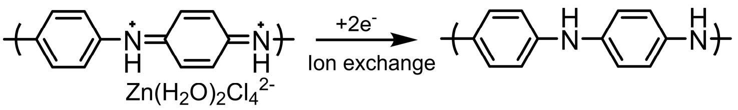

$\Delta\, m/dq = -121.7$ g mol^{-1} of e^{-}

Reaction involving cation and anion:

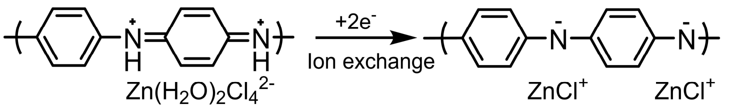

$\Delta\, m/dq = -21.8$ g mol^{-1} of e^{-}

The limitation of desolvation kinetic is also regarded as the crucial issue in the electrochemical performance at low temperature. The absence of dissociation in the ion complexes adsorption process will lead to fast kinetic and high performance of low-temperature batteries.

Supplementary References:

1. Duan, Y. et al. A point-charge force field for molecular mechanics simulations of proteins based on condensed-phase quantum mechanical calculations. *J. Comput. Chem.* **24**, 1999-2012 (2003).
2. Mo, F. et al. A flexible rechargeable aqueous zinc manganese-dioxide battery working at −20 °C. *Energy Environ. Sci.* **12**, 706-715 (2019).
3. Nian, Q. et al. Aqueous Batteries Operated at −50 °C. *Angew. Chem. Int. Ed.* **58**, 16994-16999 (2019).
4. Rustomji, C. S. et al. Liquefied gas electrolytes for electrochemical energy storage devices. *Science* **356**, eaal4263 (2017).
5. Dong, X., Guo, Z., Guo, Z., Wang, Y. & Xia, Y. Organic Batteries Operated at −70 °C. *Joule* **2**, 902-913 (2018).
6. Fan, X. et al. All-temperature batteries enabled by fluorinated electrolytes with non-polar solvents. *Nat. Energy* **4**, 882-890 (2019).
